# Transmission of COVID-19 in Nightlife, Household, and Health Care Settings in Tokyo, Japan, in 2020

**DOI:** 10.1001/jamanetworkopen.2023.0589

**Published:** 2023-02-24

**Authors:** Takeaki Imamura, Aika Watanabe, Yusuke Serizawa, Manami Nakashita, Mayuko Saito, Mayu Okada, Asamoe Ogawa, Yukiko Tabei, Yoshiko Soumura, Yoko Nadaoka, Naoki Nakatsubo, Takashi Chiba, Kenji Sadamasu, Kazuhisa Yoshimura, Yoshihiro Noda, Yuko Iwashita, Yuji Ishimaru, Naomi Seki, Kanako Otani, Tadatsugu Imamura, Matthew Myers Griffith, Kelly DeToy, Motoi Suzuki, Michihiko Yoshida, Atsuko Tanaka, Mariko Yauchi, Tomoe Shimada, Hitoshi Oshitani

**Affiliations:** 1 Department of Virology, Tohoku University Graduate School of Medicine, Sendai, Japan; 2 Itabashi-City Public Health Center, Tokyo, Japan; 3 National Defense Medical College Hospital, Saitama, Japan; 4 National Institute of Infectious Diseases, Tokyo, Japan; 5 Tokyo Metropolitan Institute of Public Health, Tokyo, Japan; 6 Public Health and Disease Prevention Division, Suginami City Public Health Center, Tokyo, Japan; 7 Department of Plastic, Reconstructive and Aesthetic Surgery, Nippon Medical School, Tokyo, Japan; 8 Tama Tachikawa Public Health Center, Tokyo, Japan; 9 Bureau of Social Welfare and Public Health, Tokyo Metropolitan Government, Tokyo, Japan; 10 Ota City Public Health Center, Tokyo, Japan; 11 National Center for Child Health and Development, Tokyo, Japan; 12 National Centre for Epidemiology and Population Health, the Australian National University, Canberra, Australia; 13 Division of Global Disease Epidemiology and Control, Department of International Health, Johns Hopkins Bloomberg School of Public Health, Baltimore, Maryland; 14 Minato Public Health Center, Tokyo, Japan; 15 Bunkyo-City Public Health Center, Tokyo, Japan

## Abstract

**Importance:**

There have been few studies on the heterogeneous interconnection of COVID-19 outbreaks occurring in different social settings using robust, surveillance epidemiological data.

**Objectives:**

To describe the characteristics of COVID-19 transmission within different social settings and to evaluate settings associated with onward transmission to other settings.

**Design, Setting, and Participants:**

This is a case series study of laboratory-confirmed COVID-19 cases in Tokyo between January 23 and December 5, 2020, when vaccination was not yet implemented. Using epidemiological investigation data collected by public health centers, epidemiological links were identified and classified into 7 transmission settings: imported, nightlife, dining, workplace, household, health care, and other.

**Main Outcomes and Measures:**

The number of cases per setting and the likelihood of generating onward transmissions were compared between different transmission settings.

**Results:**

Of the 44 054 confirmed COVID-19 cases in this study, 25 241 (57.3%) were among male patients, and the median (IQR) age of patients was 36 (26-52) years. Transmission settings were identified in 13 122 cases, including 6768 household, 2733 health care, and 1174 nightlife cases. More than 6600 transmission settings were detected, and nightlife (72 of 380 [18.9%]; *P* < .001) and health care (119 [36.2%]; *P* < .001) settings were more likely to involve 5 or more cases than dining, workplace, household, and other settings. Nightlife cases appeared in the earlier phase of the epidemic, while household and health care cases appeared later. After adjustment for transmission setting, sex, age group, presence of symptoms, and wave, household and health care cases were less likely to generate onward transmission compared with nightlife cases (household: adjusted odds ratio, 0.03; 95% CI, 0.02-0.05; health care: adjusted odds ratio, 0.57; 95% CI, 0.41-0.79). Household settings were associated with intergenerational transmission, while nonhousehold settings mainly comprised transmission between the same age group. Among 30 932 cases without identified transmission settings, cases with a history of visiting nightlife establishments were more likely to generate onward transmission to nonhousehold settings (adjusted odds ratio, 5.30 [95% CI, 4.64-6.05]; *P* < .001) than those without such history.

**Conclusions and Relevance:**

In this case series study, COVID-19 cases identified in nightlife settings were associated with a higher likelihood of spreading COVID-19 than household and health care cases. Surveillance and interventions targeting nightlife settings should be prioritized to disrupt COVID-19 transmission, especially in the early stage of an epidemic.

## Key Points

**Question:**

Which social settings are associated with large outbreaks and onward transmission of COVID-19?

**Findings:**

In this case series study that analyzed 44 054 confirmed COVID-19 cases, transmission events in nightlife and health care settings were more likely to involve 5 or more cases. Household and health care cases were significantly less likely to generate onward transmission than nightlife cases.

**Meaning:**

This case series highlights the heterogenic characteristics of social settings regarding their roles in COVID-19 transmission and the significance of nightlife settings for further COVID-19 spread.

## Teaser

This case series describes the characteristics of COVID-19 transmission within different social settings in Tokyo, Japan, and evaluates which settings are associated with onward transmission.

## Introduction

The COVID-19 pandemic has had substantial consequences worldwide. Despite an accelerated COVID-19 vaccine rollout, countries with high vaccination rates have reported a resurgence of cases due to the emergence of new variants and the waning of vaccine-induced immunity.^1^ Nonpharmaceutical interventions (NPIs) have been implemented globally,^2^ but strict NPIs reducing human activity and mobility have resulted in significant socioeconomic consequences.^3^ Therefore, NPIs must be optimized to balance public health benefits and societal consequences.^4^

One approach for balanced control measures may be targeting high-risk settings of COVID-19 spread. Most COVID-19 cases do not generate secondary cases, but a few generate many,^5^ suggesting an important role of superspreading events (SSEs) in sustained COVID-19 transmission.^6,7^ The exponential growth of COVID-19 cases implies that multiple SSEs must be connected within transmission chains.^8,9^ The potential role of multiple SSEs in sustained transmission has been outlined in severe acute respiratory syndrome and Middle East respiratory syndrome.^10,11^

Developing balanced control measures requires knowing which settings are more likely to involve many cases and generate transmission beyond that setting. For COVID-19, outbreaks have been reported in nightclubs, bars, religious gatherings, factories, dormitories, schools, hospitals, and other settings.^12^ Outbreaks in health care facilities often result in many severe cases and deaths.^13^ Household settings may be important for intergenerational mixing.^14^ Furthermore, transmission from one setting to others (ie, onward transmission) may be heterogeneous depending on the setting.^12^ Community transmission following outbreaks at restaurants, bars, and nightclubs has been reported throughout the COVID-19 pandemic.^15,16,17,18^ Previous studies on tuberculosis transmission identified bars, shelters, and places where illicit drugs are used as high-risk settings for onward transmission.^19,20^ Nevertheless, the evidence suggesting restaurants and bars as high-risk settings of onward transmission is limited to case studies. The role of health care facilities and household settings in spreading transmission to other settings and populations remains unclear. Studies using robust, surveillance epidemiological data from confirmed cases to assess the high-risk settings of large outbreaks and onward transmission are lacking.

Thus, we aimed to understand different characteristics of transmission settings and their attribution to subsequent community transmission. Specifically, we analyzed the temporal associations of cases identified in transmission settings, the number of cases involved per setting, factors associated with onward transmission, and age matrices of primary and offspring cases using data from detailed epidemiological investigations in Tokyo, Japan.

## Methods

### Design, Setting, and Participants

In this case series study, we analyzed laboratory-confirmed COVID-19 cases reported in Tokyo between January 23 and December 5, 2020, when vaccination was not yet implemented. Public health centers conducted retrospective investigations and prospective contact tracing of every confirmed case for 14 days.^21^ Epidemiological data for each case were recorded in an investigation form and then registered in the national database (eAppendix in Supplement 1). Countermeasures implemented in Japan included government recommendations to stay at home and avoid nonessential travel as well as the closure of targeted businesses and the limiting of business hours and alcohol sales at serving establishments. No mandatory lockdowns were implemented.

This study was conducted to support an ongoing public health response. Ethical approval and the requirement for informed consent for this study was waived by the Infectious Disease Control Division of the Tokyo Metropolitan Government Bureau of Social Welfare and Public Health. Data are presented following the reporting guideline for case series.^22^

### Definitions

COVID-19 cases were confirmed by laboratory diagnosis, including SARS-CoV-2 gene detection, SARS-CoV-2 isolation, and qualitative and quantitative antigen tests; double-counting was eliminated by case identifiers. We divided the study period into the 3 epidemic waves (wave 1, January 14 to May 25, 2020; wave 2, May 26 to September 30, 2020; and part of wave 3, October 1 to December 6, 2020) and assigned the cases to 1 of the 3 waves according to their dates of confirmation. Wave 3 peaked in early January 2021 and subsided at the end of February 2021. For administrative reasons, we could only include the early part of wave 3.

Epidemiological links were defined if a case had close contact with 1 or more confirmed cases, as long as the dates of onset and confirmation of cases were fewer than 14 days apart (eFigure 1 in Supplement 1). A transmission setting was defined as where an epidemiological link occurred based on reported place(s) and time(s). Cases can belong to multiple transmission settings if they are so linked. Transmission settings were classified into 7 categories: imported, nightlife, dining, workplace, household, health care, and other (unclassified) (eTable 1 in Supplement 1). The nightlife settings included restaurants, bars, nightclubs (including host and hostess clubs), and other eating and drinking establishments operating at night. The dining settings were defined as those involving eating and drinking establishments that did not fulfill the definition of nightlife settings. Health care settings included hospitals, outpatient facilities, long-term care facilities for older patients and those with disabilities, and adult day services centers. Regarding transmission in the household, we included cases likely to be infected by household members as well as those cases likely to be infected by their second degree of kinship, regardless of whether they lived in the same household.

Among cases linked in the same transmission settings, we defined the primary case as that with a prior epidemiological link with cases from other settings and defined offspring cases as the rest. If no case in a setting had a prior epidemiological link, we defined the case with the earliest date of onset or confirmation in the setting as the primary case. We did not define primary cases in health care settings because identifying COVID-19 introduction to a health care facility was unfeasible, even with detailed epidemiological investigations. An outbreak involving 5 or more offspring cases was considered a large outbreak.

We defined onward transmission as a transmission from one setting to another. When onward transmission occurred, we regarded all of the cases in that setting as offspring cases, even if multiple generations were possible in that setting (eFigure 1 in Supplement 1). Onward transmission to health care settings was not analyzed in this study.

### Statistical Analysis

Categorical and continuous variables were compared using χ^2^ tests with Šidák corrections and Kruskal-Wallis test with Dunn test, respectively. Logistic regression was used to evaluate factors associated with COVID-19–related deaths. We used generalized estimating equation models to account for the within–transmission setting association when comparing the interval between onset dates of cases and wave peaks between known transmission settings and analyzing characteristics of primary cases associated with onward transmission. For cases without known transmission settings, we compared the onset wave’s peak interval by a history of visiting nightlife establishments and the association of such history with onward transmission using logistic regression. Multivariable regression models were used to adjust for sex, age group (0-17, 18-39, 40-64, and ≥65 years), presence of symptoms, wave, and transmission settings or a history of visiting nightlife establishments. *P* < .05 was considered statistically significant, and all tests were 2-tailed. All statistical analyses were performed using Stata/IC version 15.1 (StataCorp).

## Results

Among 44 054 confirmed COVID-19 cases in this study, 25 241 (57.3%) were among male patients, and the median (IQR) patient age was 36 (26-52) years. Transmission settings were identified in 13 122 cases (29.8%). The household was the most frequently identified setting (6768 [51.6%]), followed by health care (2733 [20.8%]) (eTable 2 in Supplement 1). The proportion of cases among male individuals was the greatest in nightlife settings (823 of 1174 [70.1%]) compared with all other settings, and the differences were statistically significant (*P* < .001) except when compared with imported (97 of 152 [63.8%] among male individuals; *P* = .98) and workplace (456 of 702 [65.0%]; *P* = .53) settings (Table 1). Cases in other settings, which included cases identified in nurseries, schools, and universities, had the youngest median (IQR) patient age (24 [20-36] years; *P* < .001 vs all other settings), followed by nightlife settings (29 [24-39]; *P* < .001 vs imported, dining, workplace, household, health care, and unknown settings). The median (IQR) number of days from onset to confirmation was the longest in imported settings (6 [4-9] days; *P* < .001 vs all other settings), followed by nightlife (5 [3-8]; *P* < .001 vs dining, workplace, household, health care, other, and unknown settings). The case fatality ratio (CFR) was the largest in health care settings (268 of 2733 [9.8%]; *P* < .001). All fatal cases in health care settings occurred among patients or visitors (ie, none among health care workers). The CFR significantly decreased from wave 1 (332 of 5173 [6.4%]) to wave 2 (127 of 20 858 [0.6%]; *P* < .001) (eTable 2 in Supplement 1). The CFR of wave 3 (104 of 18 023 [0.6%]) might be underestimated because our data only included the first 2 months of the wave 3 period. The proportion of older adults (aged ≥65 years) and the number of days from onset to confirmation decreased from wave 1 to wave 2 and 3, while the proportion of asymptomatic cases significantly increased.

**Table 1.** Characteristics of 44 054 COVID-19 Cases by Different Virus Transmission Settings

Characteristic	Cases, No. (%)								
	Total (N = 44 054)	Imported (n = 152)	Nightlife (n = 1174)	Dining (n = 275)	Workplace (n = 702)	Household (n = 6768)	Health care (n = 2733)	Other (n = 1318)	Unknown (n = 30 932)
Sex									
Male^a^	25 241 (57.3)	97 (63.8)	823 (70.1)	153 (55.6)	456 (65.0)	2754 (40.7)	1018 (37.2)	784 (59.5)	19 156 (61.9)
Female	18 813 (42.7)	55 (36.2)	351 (29.9)	122 (44.4)	246 (35.0)	4014 (59.3)	1715 (62.8)	534 (40.5)	11 776 (38.1)
Age, y									
Median (IQR)^a^	36 (26-52)	42 (26-56)	29 (24-39)	34 (27-44)	37 (27-50)	40 (24-57)	65 (35-83)	24 (20-36)	36 (26-50)
0-17^a^	1802 (4.1)	10 (6.6)	3 (0.3)	6 (2.2)	0 (0.0)	1129 (16.7)	14 (0.5)	166 (12.6)	474 (1.5)
18-39	22 833 (51.8)	64 (42.1)	891 (75.9)	179 (65.1)	385 (54.8)	2245 (33.2)	775 (28.4)	875 (66.4)	17 419 (56.3)
40-64	13 749 (31.2)	59 (38.8)	233 (19.8)	82 (29.8)	264 (37.6)	2241 (33.1)	559 (20.5)	210 (15.9)	10 101 (32.7)
≥65	5670 (12.9)	19 (12.5)	47 (4.0)	8 (2.9)	53 (7.5)	1153 (17.0)	1385 (50.7)	67 (5.1)	2938 (9.5)
Symptoms									
Symptomatic^a^	37 150 (84.3)	131 (86.2)	940 (80.1)	218 (79.3)	441 (62.8)	4790 (70.8)	1988 (72.7)	957 (72.6)	27 685 (89.5)
Asymptomatic	6904 (15.7)	21 (13.8)	234 (19.9)	57 (20.7)	261 (37.2)	1978 (29.2)	745 (27.3)	361 (27.4)	3247 (10.5)
Waves									
1^a^	5173 (11.7)	123 (80.9)	122 (10.4)	13 (4.7)	64 (9.1)	683 (10.1)	1069 (39.1)	58 (4.4)	3041 (9.8)
2	20 858 (47.3)	8 (5.3)	900 (76.7)	169 (61.5)	324 (46.2)	2832 (41.8)	697 (25.5)	570 (43.2)	15 358 (49.7)
3	18 023 (40.9)	21 (13.8)	152 (12.9)	93 (33.8)	314 (44.7)	3253 (48.1)	967 (35.4)	690 (52.4)	12 533 (40.5)
Time from onset to confirmation, median (IQR), d									
Overall^a^	4 (2-7)	6 (4-9)	5 (3-8)	4 (2-6)	4 (2-6)	3 (2-6)	3 (1-6)	3 (2-5)	4 (3-7)
Wave									
1^a^	8 (5-10)	7 (5-10)	8 (6-12)	10 (7-14)	8 (6-10)	7 (4-9)	5 (3-8)	9 (6-11)	8 (6-11)
2^a^	4 (3-6)	5 (4-6)	5 (3-7)	4 (3-6)	3 (2-5)	3 (2-5)	2 (1-4)	3 (2-6)	4 (3-7)
3^a^	3 (2-5)	3 (2-5)	3 (2-5)	3 (2-5)	3 (2-6)	3 (1-4)	1 (0-3)	3 (1-4)	3 (2-6)
Outcome									
Died^a^	563 (1.3)	3 (2.0)	3 (0.3)	0	2 (0.3)	58 (0.9)	268 (9.8)	3 (0.2)	226 (0.7)
Survived	43 491 (98.7)	149 (98.0)	1171 (99.7)	275 (100)	700 (99.7)	6710 (99.1)	2465 (90.2)	1315 (99.8)	30 706 (99.3)
Cases generating onward transmission									
No^a^	38 149 (86.6)	134 (88.2)	1068 (91.0)	252 (91.6)	624 (88.9)	6732 (99.5)	2563 (93.8)	1167 (88.5)	25 609 (82.8)
Yes	5905 (13.4)	18 (11.8)	106 (9.0)	23 (8.4)	78 (11.1)	36 (0.5)	170 (6.2)	151 (11.5)	5323 (17.2)
Onward transmission toward									
Only household^a^	4634 (10.5)	16 (10.5)	80 (6.8)	18 (6.5)	75 (10.7)	NA	169 (6.2)	136 (10.3)	4140 (13.4)
Only nonhousehold	1098 (2.5)	2 (1.3)	25 (2.1)	3 (1.1)	2 (0.3)	36 (0.5)	1 (0.0)	13 (1.0)	1016 (3.3)
Both household and non-household	173 (0.4)	0	1 (0.1)	2 (0.7)	1 (0.1)	NA	0	2 (0.2)	167 (0.5)

Abbreviation: NA, not applicable.

^a^ *P* < .001. The Kruskal-Wallis and the χ^2^ tests were used to evaluate differences between 8 transmission settings regarding continuous and categorical variables, respectively.

When compared with household cases, the adjusted odds ratio (aOR) of death for health care cases was higher (aOR, 3.32 [95% CI, 2.45-4.51]; *P* < .001) (eTable 3 in Supplement 1). Other factors statistically associated with death were male sex (aOR, 1.90 [95% CI, 1.57-2.31]; *P* < .001) and age 65 years or older (aOR, 39.85 [95% CI, 30.05-52.85]; *P* < .001). Compared with wave 1 cases, wave 2 and 3 cases were less likely to result in deaths.

Among 13 122 cases with identified transmission settings, 12 970 cases (excluding imported) were identified in 6624 transmission settings occurring in Japan (eTable 4 in Supplement 1). Nightlife (72 of 380 [18.9%]; *P* < .001) and health care (119 [36.2%]; *P* < .001) settings were associated with a higher probability of involving 5 or more offspring cases than dining, workplace, household, and other settings. Nightlife settings with hosts or hostesses (59 of 193 [30.6%]) more frequently involved 5 or more offspring cases compared with those without hosts or hostesses (13 of 187 [7.0%]; *P* < .001).

Among 6624 settings, 6121 primary cases were identified, excluding unidentified primary cases of health care settings and cases without detailed information. Of these, 176 (2.9%) generated 5 or more offspring cases. Although the previous transmission settings were not known (unknown) in 167 (94.9%) of those (eTable 4 in Supplement 1), the proportion of cases generating 5 or more offspring cases was larger in primary cases who visited nightlife establishments than those who did not (72 of 712 [10.1%] vs 95 of 4817 [2.0%]; *P* < .001). Cases among patients aged 18 to 39 years (125 of 2686 [4.7%]) had a significantly larger proportion of primary cases generating 5 or more offspring cases than primary cases among patients aged 40 to 64 years (28 of 2307 [1.2%]; *P* < .001) and 65 years or older (13 of 918 [1.4%]; *P* < .001). Moreover, cases taking 4 or more days from onset to confirmation (122 of 3079 [4.0%]; *P* = .003) had a larger proportion of primary cases generating 5 or more offspring cases than those taking 3 or fewer days.

**Figure 1.**
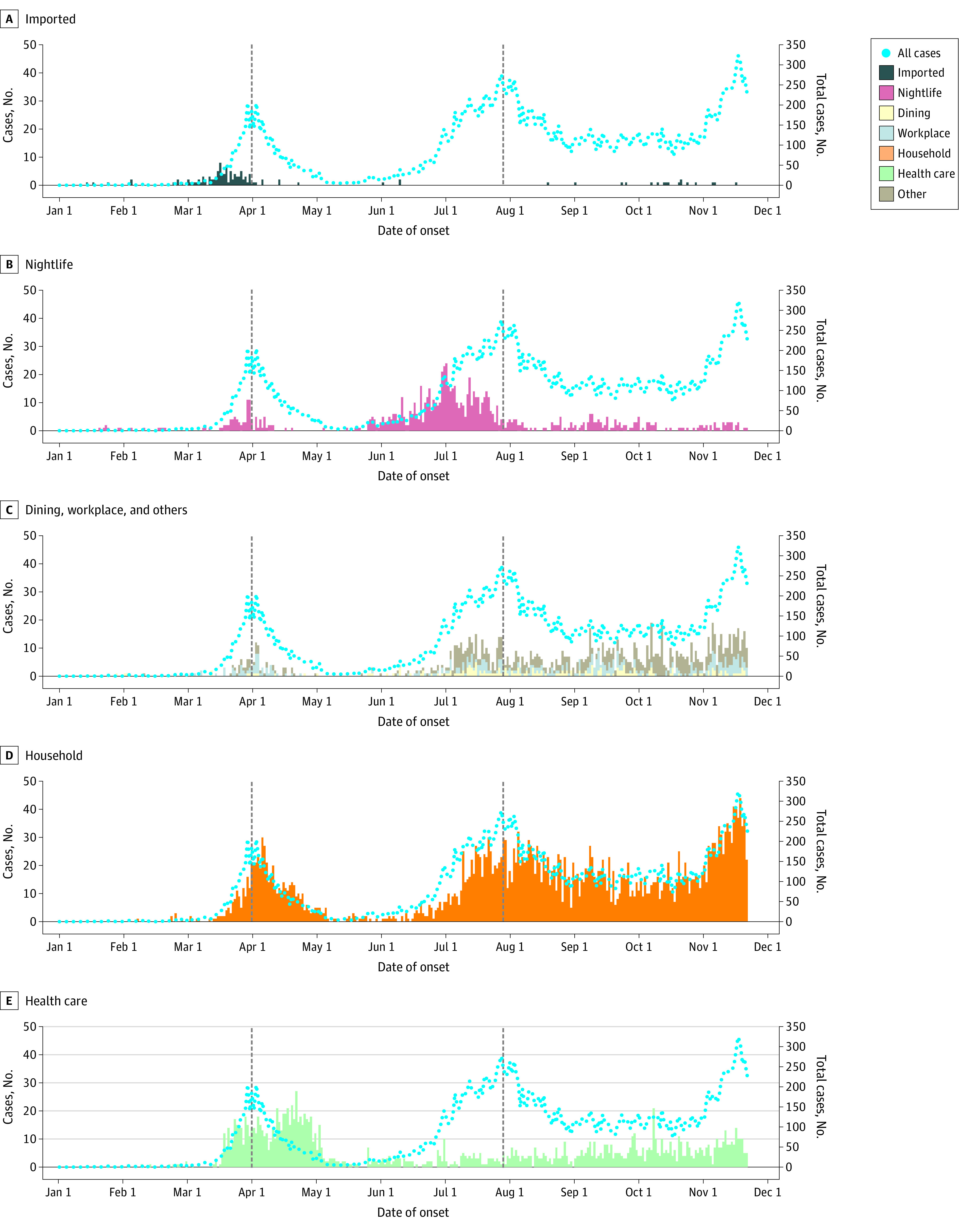
Daily Number of COVID-19 Cases by Different Transmission Settings Based on Date of Onset The daily number of cases identified from imported, nightlife, dining, workplace, other, household, and health care settings based on the date of onset are shown using bars. The blue dots indicate the daily number of all cases, including unknown cases. Vertical dashed lines indicate the peak of wave 1 (April 1, 2020) and wave 2 (July 29, 2020), which was determined as the date with the largest 7-day moving average daily number of cases in respective waves.

**Figure 2.**
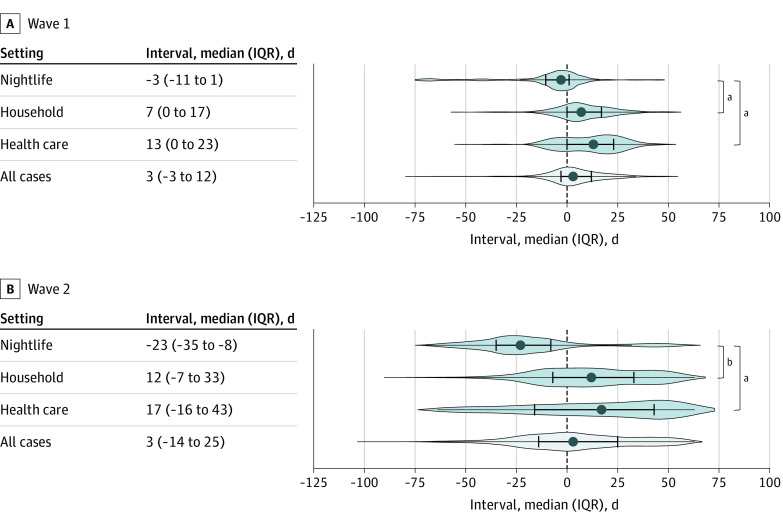
Comparison of the Interval Between Each Case’s Date of Onset and the Respective Wave’s Peak by Different Transmission Settings The interval between each case's date of onset and the respective wave's peak (wave 1, April 1, 2020; wave 2, July 29, 2020) was calculated. The distribution of intervals was plotted regarding nightlife, household, and health care settings, as well as all the cases including unknown cases, stratified by wave 1 and wave 2. The distribution of intervals between nightlife, household, and health care cases were compared using the generalized estimating equations with Šidák corrections to account for the within-cluster association. ^a^*P* < .05. ^b^*P* < .001.

Cases identified in nightlife settings occurred in the earlier phase of waves 1 and 2, while household and health care cases appeared in the later phase of both waves (Figure 1 and Figure 2). In Wave 1, the median date of onset of household and health care cases was 10 and 16 days later, respectively, than that of nightlife cases (Figure 2). In wave 2, the difference was 35 and 40 days for household and health care cases, respectively. The daily number of health care cases in wave 1 displayed a bimodal distribution with peaks in late March and late April (Figure 1). In March, a single hospital SSE caused by the ancestral strain resulted in more than 200 cases, while the strain with D614G substitution in the spike protein caused health care SSEs in April.^23,24^

Of 13 122 cases with identified transmission settings, 582 cases (4.4%) generated onward transmission to other settings (Table 1). The proportion of cases generating onward transmission was the smallest in household settings, significantly lower than nightlife cases by univariable analysis (eTable 5 in Supplement 1). Furthermore, male sex, symptomatic infection, and taking 4 or more days from onset to confirmation were positively associated with the generation of onward transmission, while cases in wave 3 were less likely to generate onward transmission than wave 1 cases. After adjusting for transmission setting, sex, age group, presence of symptoms, and wave, cases in household (aOR, 0.03 [95% CI, 0.02-0.05]; *P* < .001) and health care (aOR, 0.57 [95% CI, 0.41-0.79]; *P* = .001) settings were significantly less likely to generate onward transmission compared with cases in nightlife settings (Table 2). Symptomatic cases were more likely to generate onward transmission (aOR, 1.87 [95% CI, 1.49-2.34]; *P* < .001). Despite the nonsignificant differences between age groups in univariable analysis, cases in 3 other age groups (0-17, 40-64, and ≥65 years) were significantly more likely to generate onward transmission compared with cases aged 18-39 years (0-17 years: aOR, 4.33 [95% CI, 3.03-6.18]; *P* < .001; 40-64 years: aOR, 1.82 [95% CI, 1.46-2.27]; *P* < .001; ≥65 years: aOR, 2.03 [95% CI, 1.56-2.66]; *P* < .001) as revealed by multivariable analysis. Among 1174 nightlife cases, the proportion of cases generating onward transmission was smaller in individuals aged 18 to 39 years (63 of 891 [7.1%]) compared with those aged 40 to 64 years (36 of 223 [15.5%]) and 65 years or older (7 of 47 [14.9%]). Unlike in univariable analysis, sex and wave were no longer associated with onward transmission in multivariable analysis.

**Table 2.** Association of Transmission Settings and Other Factors With the Generation of Total OWT, Toward Nonhousehold Settings, and Toward the Household Setting^a^

Characteristic	OWT											
	Total				Toward nonhousehold				Toward household			
	No. (%)		aOR (95%CI)	*P* value	No. (%)		aOR (95%CI)	*P* value	No. (%)		aOR (95%CI)	*P* value
	Without OWT^b^	With OWT^c^			Without OWT^b^	With OWT^c^			Without OWT^d^	With OWT^e^		
Cases	12 540 (95.56)	582 (4.44)	NA	NA	13 034 (99.33)	88 (0.67)	NA	NA	5854 (92.13)	500 (7.87)	NA	NA
Transmission setting												
Imported	134 (88.16)	18 (11.84)	1.06 (0.59-1.90)	.84	150 (98.68)	2 (1.32)	0.71 (0.15-3.36)	.67	136 (89.47)	16 (10.53)	1.11 (0.60-2.06)	.75
Nightlife	1068 (90.97)	106 (9.03)	1 [Reference]	NA	1148 (97.79)	26 (2.21)	1 [Reference]	NA	1093 (93.10)	81 (6.90)	1 [Reference]	NA
Dining	252 (91.64)	23 (8.36)	0.84 (0.52-1.36)	.48	270 (98.18)	5 (1.82)	0.78 (0.30-2.07)	.62	255 (92.73)	20 (7.27)	0.95 (0.56-1.60)	.84
Workplace	624 (88.89)	78 (11.11)	1.24 (0.87-1.76)	.23	699 (99.57)	3 (0.43)	0.22 (0.07-0.74)	.01	626 (89.17)	76 (10.83)	1.57 (1.08-2.28)	.02
Household	6732 (99.47)	36 (0.53)	0.03 (0.02-0.05)	<.001	6732 (99.47)	36 (0.53)	0.23 (0.13-0.42)	<.001	NA	NA		
Health care	2563 (93.78)	170 (6.22)	0.57 (0.41-0.79)	.001	2732 (99.96)	1 (0.04)	0.02 (0.00-0.15)	<.001	2564 (93.82)	169 (6.18)	0.69 (0.48-0.99)	.04
Other	1167 (88.54)	151 (11.46)	1.10 (0.81-1.49)	.55	1303 (98.86)	15 (1.14)	0.51 (0.26-0.99)	.05	1180 (89.53)	138 (10.47)	1.21 (0.86-1.70)	.27
Sex												
Female	6754 (95.98)	283 (4.02)	1 [Reference]	NA	6993 (99.37)	44 (0.63)	1 [Reference]	NA	2781 (91.99)	242 (8.01)	1 [Reference]	NA
Male	5786 (95.09)	299 (4.91)	0.91 (0.77-1.09)	.32	6041 (99.28)	44 (0.72)	0.82 (0.53-1.27)	.38	3073 (92.25)	258 (7.75)	0.93 (0.77-1.13)	.47
Age group, y												
0-17	1264 (95.18)	64 (4.82)	4.33 (3.03-6.18)	<.001	1318 (99.25)	10 (0.75)	1.56 (0.74-3.25)	.24	145 (72.86)	54 (27.14)	5.66 (3.77-8.50)	<.001
18-39	5200 (96.05)	214 (3.95)	1 [Reference]	NA	5368 (99.15)	46 (0.85)	1 [Reference]	NA	2999 (94.64)	170 (5.36)	1 [Reference]	NA
40-64	3483 (95.48)	165 (4.52)	1.82 (1.46-2.27)	<.001	3623 (99.31)	25 (0.69)	1.16 (0.69-1.93)	.58	1264 (89.84)	143 (10.16)	2.00 (1.57-2.55)	<.001
≥65	2593 (94.91)	139 (5.09)	2.03 (1.56-2.66)	<.001	2725 (99.74)	7 (0.26)	0.85 (0.37-1.97)	.70	1446 (91.58)	133 (8.42)	2.32 (1.74-3.09)	<.001
Symptoms												
Asymptomatic	3551 (97.10)	106 (2.90)	1 [Reference]	NA	3649 (99.78)	8 (0.22)	1 [Reference]	NA	1580 (94.10)	99 (5.90)	1 [Reference]	NA
Symptomatic	8989 (94.97)	476 (5.03)	1.87 (1.49-2.34)	<.001	9385 (99.15)	80 (0.85)	3.67 (1.75-7.67)	.001	4274 (91.42)	401 (8.58)	1.70 (1.34-2.16)	<.001
Wave												
1	2031 (95.26)	101 (4.74)	1 [Reference]	NA	2124 (99.62)	8 (0.38)	1 [Reference]	NA	1355 (93.51)	94 (6.49)	1 [Reference]	NA
2	5229 (95.07)	271 (4.93)	1.16 (0.86-1.56)	.33	5449 (99.07)	51 (0.93)	1.54 (0.68-3.46)	.30	2447 (91.72)	221 (8.28)	1.05 (0.77-1.44)	.76
3	5280 (96.17)	210 (3.83)	1.07 (0.79-1.44)	.68	5461 (99.47)	29 (0.53)	1.38 (0.59-3.21)	.45	2052 (91.73)	185 (8.27)	1.03 (0.74-1.42)	.88

Abbreviations: aOR, adjusted odds ratio; NA, not applicable; OWT, onward transmission.

^a^ Factors associated with the generation of OWT were compared using generalized estimating equation models for accounting within-transmission setting associations, adjusted for transmission setting, sex, age group, presence of symptoms, and wave. Nightlife setting, female sex, age 18 to 39 years, asymptomatic cases, and wave 1 were set as references. Household cases were excluded from the analysis of OWT toward household settings since household cases do not generate OWT toward household settings.

^b^ Cases not generating OWT.

^c^ Cases generating OWT.

^d^ Cases not generating OWT (excluding household cases).

^e^ Cases generating OWT (excluding household cases).

We analyzed the generation of onward transmission to household and nonhousehold settings separately. Compared with nightlife cases, workplace, household, health care, and other cases were significantly less likely to generate onward transmission to nonhousehold settings (Table 2). Compared with nightlife cases, the likelihood of generating onward transmission to household settings varied between settings: workplace cases were more likely to generate onward transmission to household settings (aOR, 1.57 [95% CI, 1.08-2.28]; *P* = .02), health care cases were less likely to (aOR, 0.69 [95% CI, 0.48-0.99]; *P* = .04), and other settings revealed no significant differences. Age group was associated with the likelihood of generating onward transmission toward household settings but not toward nonhousehold settings. Symptomatic cases were more likely to generate onward transmission both toward household and nonhousehold settings, but sex and wave were not associated with onward transmission toward either.

Regarding 30 932 cases without identified transmission settings (unknown cases), the peak of cases with a history of visiting nightlife establishments significantly preceded those without such history both in wave 1 and wave 2 (eFigure 2 in Supplement 1). Compared with unknown cases without a history of visiting nightlife establishments, unknown cases with such history were more likely to generate onward transmission to nonhousehold settings (aOR, 5.30 [95% CI, 4.64-6.05]; *P* < .001) and less likely to generate onward transmission to household settings (aOR, 0.85 [95% CI, 0.75–0.97]; *P* = .01) (eTable 6 in Supplement 1).

**Figure 3.**
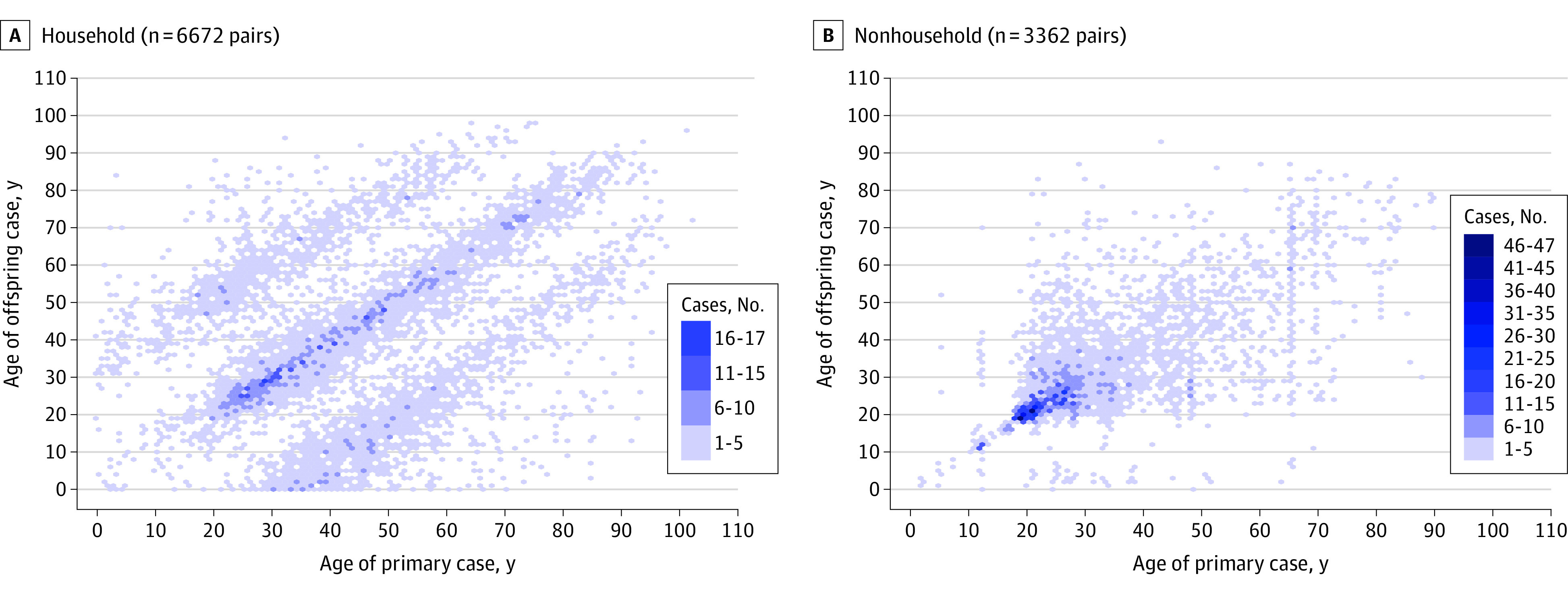
Age Distribution of Offspring Cases and Their Primary Cases The age distribution of 6672 pairs of primary and offspring cases involved in household settings (A) and 3362 pairs of primary and offspring cases involved in nonhousehold settings (B) was plotted.

The age combination of 6672 pairs of primary and offspring cases identified in household settings revealed 3 diagonal accumulations, indicating transmission between the same age groups and intergenerational transmission (Figure 3A). The age combination of 3362 pairs from nonhousehold settings revealed a single diagonal accumulation, representing transmission between cases of the same age groups (Figure 3B). Transmission to individuals aged 65 years or older occurred more frequently in household settings (1137 pairs [17.0%]) than in nonhousehold settings (170 pairs [5.1%]; *P* < .001). Intergenerational transmission from younger primary cases to older offspring cases (≥20-year difference) occurred more frequently in household settings (1166 pairs [17.5%]) compared with nonhousehold settings (163 pairs [4.8%]; *P* < .001).

## Discussion

This study described the heterogenous characteristics of COVID-19 transmission among cases linked with transmission settings and their association with further COVID-19 spread. The temporal distribution of cases favored nightlife settings early and household and health care settings later in both waves 1 and 2. Transmission in nonhousehold settings, including nightlife, mainly occurred among younger populations, while in household settings we observed intergenerational transmission to older individuals. Studies suggested that younger populations pose a high risk of generating large outbreaks^25^ and contribute to the sustained transmission of COVID-19.^8,26^ Our study also showed a large number of outbreaks with 5 or more cases and onward transmission in cases among younger individuals. However, the likelihood of generating onward transmission toward nonhousehold settings was similar among age groups. The likelihood of generating onward transmission toward household settings was, in fact, smaller in cases among younger vs older individuals, possibly reflecting the proportion of cases living with family members among age groups. Although primary cases in nightlife and health care settings were associated with involving 5 or more offspring cases, cases identified in nightlife settings were more likely to generate onward transmission than health care cases. Household settings rarely involved many cases, and the likelihood of generating onward transmission was small. Taken together, these findings suggest (1) that nightlife settings are associated with an increased likelihood of spreading COVID-19 and (2) that although household and health care settings affect populations with an increased risk of death, they are less likely to generate onward transmission.

Our finding on the potential role of nightlife settings in COVID-19 transmission seems valid. Studies using human mobility data and internet search trends indicated that the reopening of full-service restaurants and queries related to bars and restaurants preceded the increase in COVID-19 cases, suggesting that increased visits to such establishments was associated with the COVID-19 spread.^27,28^ Measures targeting nightlife settings may be an effective option for balanced interventions to control COVID-19. Closure of nightlife establishments is reportedly effective in suppressing COVID-19.^2^ Conversely, governmental schemes to promote eating and drinking at nightlife establishments preceded COVID-19 spread.^29,30^ Major improvements in the COVID-19 surveillance system in Tokyo occurred between wave 1 and 2, illustrated by the increased proportion of asymptomatic cases. Transmission chains in both waves revealed the early preponderance of nightlife cases. The COVID-19 introduction from international locations to Tokyo might have occurred more frequently in nightlife settings than others, especially in wave 1. Between waves 1 and 2, COVID-19 might have been maintained in nightlife settings, and lifting the state of emergency in May 2020 probably facilitated the resurgence of cases in nightlife settings. We did not observe a surge in nightlife cases in wave 3 of our study, which may be related to implementing control measures in nightlife establishments (eAppendix in Supplement 1). Alternatively, this finding may account for an underdetection of nightlife cases, diversification of transmission pathways, or increased cases without identified transmission settings due to incomplete contact tracing. COVID-19 outbreaks at nightlife establishments, such as end-of-year and new-year parties, were reported in Tokyo in 2020 to 2021,^30^ which was not included in our study period.

Nightlife settings are probably associated with COVID-19 transmission for multiple reasons. Crowded places, inadequate ventilation, on-site eating and drinking with frequent alcohol consumption, and aerosol- and droplet-producing behaviors, such as talking loudly and singing, can set the conditions for large outbreaks.^31,32,33^ Increased generation of onward transmission from nightlife settings may be due to social mixing patterns^34^ and sociobehavioral characteristics such as risk-taking,^35,36^ which was not addressed in this study.

The diversification of transmission pathways is accelerated with the Omicron variant circulation, including an increased incidence among children.^37,38,39^ Coupled with the vaccine roll-out, the impact of nightlife settings on COVID-19 spread might be disregarded. However, the vaccine efficacy against infection is short term,^40^ and NPIs are relaxed. The subsequent community transmission following outbreaks in nightlife settings with the Omicron variant is still reported.^17,18^ Therefore, we believe that the association of nightlife settings with COVID-19 spread is still valid.

Even with the low risk of large outbreaks and onward transmission, household settings cannot be dismissed. The large number of household cases compared with other settings can outweigh those low risks. In our study, more household cases generated onward transmission to nonhousehold settings than nightlife cases. Furthermore, because of intergenerational transmission in households, onward transmission originating from household settings may mean increased risk of infection among older patients, who have a higher risk of death. Communities with relaxed NPIs should take this concern seriously, especially with the expected increase of emerging variants with a high household secondary attack rate.^41^ Interrupting transmission chains before reaching households should be an aim of the targeted COVID-19 suppression strategy.

### Limitations

This study has limitations, including the underestimation of the number of nightlife cases and the overestimation of the number of unknown cases due to epidemiological investigations based on self-reporting and the overwhelmed capacity of public health centers, especially during periods with a surging number of cases. Characteristics might differ between cases with identified transmission settings and unknown cases given that large outbreaks facilitated the identification of transmission settings. The number of cases included in this study was limited to approximately one-quarter of estimated infection-experienced cases based on a seroprevalence study in December 2020.^42^ This study was conducted before vaccination implementation and the emergence of the Omicron variant.

## Conclusions

This case series study found an association of nightlife settings with an increased likelihood of spreading COVID-19 in Tokyo in 2020. Surveillance and interventions targeting nightlife settings should be prioritized to disrupt COVID-19 transmission chains and prevent them from reaching populations with a high risk of death, especially in the early stage of a resurgence.

## Notes

Supplement 1. **eAppendix. **Surveillance and Contact Tracing for COVID-19 in Tokyo and Areas Throughout Japan in 2020 **eFigure 1.** Concept of COVID-19 Transmission Settings and Onward Transmission **eTable 1.** Definition of Cases According to Their Identified Transmission Settings **eTable 2.** Characteristics of 44 054 COVID-19 Cases by Waves in Tokyo Between January and December 2020 **eTable 3.** Association Between COVID-19–Related Death and Characteristics of Cases **eTable 4.** Number of Transmission Settings and Characteristics of their Primary Cases by the Number of Offspring Cases per Setting **eTable 5.** Association of Transmission Settings and Other Factors With the Generation of Total Onward Transmission, to Nonhousehold Settings, and to Household Settings: Univariable Analysis **eFigure 2.** Comparison of the Interval Between Each Unknown Case’s Date of Onset and the Respective Wave’s Peak by the History of Visiting Nightlife Establishments **eTable 6.** Association of the History of Visiting Nightlife Establishments and Other Factors With the Generation of Total Onward Transmission, to Nonhousehold Settings, and to Household Settings Among Unknown Cases: A Multivariable Analysis

Supplement 2.
Data Sharing Statement
