# Supplementary material for: Transmission of COVID-19 in Nightlife, Household, and Health Care Settings in Tokyo, Japan, in 2020
Source: JAMA Netw Open. 2023 Feb 24;6(2):e230589. doi: 10.1001/jamanetworkopen.2023.0589 (PMC9958531; doi:10.1001/jamanetworkopen.2023.0589)
Supplement: Supplement 1. — eAppendix. Surveillance and Contact Tracing for COVID-19 in Tokyo and Areas Throughout Japan in 2020 eFigure 1. Concept of COVID-19 Transmission Settings and Onward Transmission eTable 1. Definition of Cases According to Their Identified Transmission Settings eTable 2. Characteristics of 44 054 COVID-19 Cases by Waves in Tokyo Between January and December 2020 eTable 3. Association Between COVID-19–Related Death and Characteristics of Cases eTable 4. Number of Transmission Settings and Characteristics of their Primary Cases by the Number of Offspring Cases per Setting eTable 5. Association of Transmission Settings and Other Factors With the Generation of Total Onward Transmission, to Nonhousehold Settings, and to Household Settings: Univariable Analysis eFigure 2. Comparison of the Interval Between Each Unknown Case’s Date of Onset and the Respective Wave’s Peak by the History of Visiting Nightlife Establishments eTable 6. Association of the History of Visiting Nightlife Establishments and Other Factors With the Generation of Total Onward Transmission, to Nonhousehold Settings, and to Household Settings Among Unknown Cases: A Multivariable Analysis [file jamanetwopen-e230589-s001.pdf]

Supplemental Online Content

Imamura T, Watanabe A, Serizawa Y, et al. Transmission of COVID-19 in nightlife, household, and health care settings in Tokyo, Japan, in 2020. *JAMA Netw Open.* 2023;6(2):e230589.
doi:10.1001/jamanetworkopen.2023.0589

eAppendix. Surveillance and Contact Tracing for COVID-19 in Tokyo and Areas Throughout Japan in 2020

eFigure 1. Concept of COVID-19 Transmission Settings and Onward Transmission

eTable 1. Definition of Cases According to Their Identified Transmission Settings

eTable 2. Characteristics of 44 054 COVID-19 Cases by Waves in Tokyo Between January and December 2020

eTable 3. Association Between COVID-19–Related Death and Characteristics of Cases

eTable 4. Number of Transmission Settings and Characteristics of their Primary Cases by the Number of Offspring Cases per Setting

eTable 5. Association of Transmission Settings and Other Factors With the Generation of Total Onward Transmission, to Nonhousehold Settings, and to Household Settings: Univariable Analysis

eFigure 2. Comparison of the Interval Between Each Unknown Case's Date of Onset and the Respective Wave's Peak by the History of Visiting Nightlife Establishments

eTable 6. Association of the History of Visiting Nightlife Establishments and Other Factors With the Generation of Total Onward Transmission, to Nonhousehold Settings, and to Household Settings Among Unknown Cases: A Multivariable Analysis

This supplemental material has been provided by the authors to give readers additional information about their work.

© 2023 Imamura T et al. *JAMA Network Open.*

eAppendix. Surveillance and Contact Tracing for COVID-19 in Tokyo and Areas Throughout Japan in 2020

The Infectious Diseases Act mandated reporting all suspected and confirmed COVID-19 cases in Japan starting January 6th, 2020. Public health officials interviewed everyone who tested positive for SARS-CoV-2 and identified contacts who would require quarantine and testing. This included conducting backward contact tracing to assess potential infectious sources within 14 days prior to symptom onset or confirmation, as well as forward contact tracing for 14 days.[1,2] Epidemiological data of each case were recorded in national databases; the National Epidemiological Surveillance of Infectious Disease,[3] and later in the Health Center Real-time Information-sharing System on COVID-19.[4]

The first COVID-19 case was detected in Japan on January 16th, 2020. On January 28th, 2020, the Act on the Prevention of Infectious Diseases and Medical Care for Patients with Infectious Diseases (Infectious Diseases Control Law) specified COVID-19 as a designated infectious disease. COVID-19 was defined as an infectious disease subject to quarantine measures by the Quarantine Infectious Diseases Act, which required mandatory reporting and isolation of all COVID-19 cases and quarantining of their contacts. COVID-19 cases were initially confirmed by detecting the SARS-CoV-2 RNA via PCR or by culturing SARS-CoV-2 from clinical specimens.[5] The National Institute for Infectious Diseases and Local Public Health Institute conducted all laboratory testing for COVID-19 in January and February. In March 2020, the Ministry of Health, Labour and Welfare extended medical insurance coverage to PCR testing, which enabled medical institutions to send samples for PCR testing to private laboratories, thereby significantly increasing the country's testing capacity. The Ministry of Health, Labour and Welfare approved various other confirmatory tests, including qualitative and quantitative antigen tests, in May and June 2020.[5]

Public Health Response in Tokyo and Areas throughout Japan in 2020

In Japan, there are no laws restricting the activities of individuals or companies with regard to public health measures. Although many countries implemented mobility restrictions in the early stages of the COVID-19 epidemic, the Japanese government requested its people to reduce human contact to control the COVID-19 spread, but these measures were optional and left to the discretion of the individual.[6] Tokyo is the capital of Japan, with an approximate population of 14 million as of October 1st, 2020, constituting 10% of Japan's total population. Furthermore, approximately 2.9 million people commute to Tokyo daily from neighboring prefectures for work or school. The first COVID-19 case in Japan was reported on January 16th in a prefecture next to Tokyo, and the first COVID-19 case in Tokyo was detected on January 23rd. The Tokyo Metropolitan Government (TMG) set up the Tokyo Metropolitan Emergency Management Council on Novel Coronavirus-Related Pneumonia on the same day. The TMG called for cooperation in COVID-19 control. The mobility restrictions employed by TMG – such as a request for remote work, stay-at-home, and canceling group events – also affected neighboring prefectures in the commuting area. These measures were issued as requests to citizens and companies in Tokyo, with no penalties for ignoring the restrictions.

© 2023 Imamura T et al. *JAMA Network Open.*

The measures deployed by TMG are listed in chronological order (eText Table), which included a request for avoiding the "3 C's" ("Closed spaces with poor ventilation," "Crowded spaces with many people nearby," and "Close-contact settings such as close-range conversations"), business closures, limiting business hours, cancellation of mass gathering events, restricting event sizes and limiting seating capacity, encouraging remote work, and strengthening healthcare and PCR testing capacity. The first COVID-19 superspreading event (SSE) detected in Tokyo was a New Year's party conducted on a party boat. In a press conference on March 30th, the Tokyo Governor requested citizens of Tokyo to refrain from visiting nightlife establishments. During the State of Emergency period, from April 11th to May 25th, restaurants and bars were requested to limit their business hours to between 5 am and 8 pm (excluding carryout and deliveries). After lifting the State of Emergency, the TMG continued requesting restaurants and bars to limit their business hours based on their roadmap of stepwise relaxation of infection control measures until June 18th. Early in the second surge in June, many SSEs were reported in Shinjuku and Ikebukuro, where large nightlife districts in Tokyo are located. The nightlife establishments with hosts and hostesses in those districts were connected to many reported SSEs. As a reaction, the Tokyo Governor publicly advised against visiting nightlife establishments (e.g., restaurants, bars, nightclubs with hosts and hostesses) in those areas. In the Shinjuku district, mass screening and more robust infection control measures were implemented, with active cooperation from owners and employees of nightlife establishments. On July 22nd, the Japanese government began a nationwide campaign to promote domestic travel ("Go to Travel" campaign), excluding Tokyo. On October 1st, the Japanese government started a campaign to promote dining out ("Go to Eat" campaign) and expanded the "Go to Travel" campaign to include Tokyo. The TMG also commenced its own campaign to promote travel within Tokyo ("Motto Tokyo" campaign) later in October. Both the "Go to Eat" and "Motto Tokyo" campaigns, however, were canceled by the end of November due to the resurgence of COVID-19 cases (Wave 3), and the "Go to Travel" campaign was suspended in December. Throughout 2020, the TMG intermittently requested business closures or limiting-business-hour of restaurants and bars in April, May, June, August, September, November, and December.

eText Table. Timeline of COVID-19-related events, measures, and announcements in 2020.

Date	International and Japanese Events and announcements	Events, measures, and announcements by the Tokyo Metropolitan Government (TMG)	URL
05-Jan-20	○ WHO China Country Office reports 44 cases of pneumonia of unknown etiology in Wuhan, China		https://www.who.int/emergencies/disease-outbreak-news/item/2020-DON229 (English)
06-Jan-20	●MHLW announcement calling for investigation of the atypical pneumonia outbreak in Wuhan		https://www.mhlw.go.jp/content/10900000/000582709.pdf (Japanese)
09-Jan-20	○WHO is informed that coronavirus is a possible pathogen causing the cluster of pneumonia cases		https://www.who.int/china/news/detail/09-01-2020-who-statement-regarding-cluster-of-pneumonia-cases-in-wuhan-china (English)
11~12-Jan-20	○China CDC publicly shares the gene sequence of the novel coronavirus 2019		https://web.archive.org/web/20200114084712/https://www.cdc.gov/coronavirus/novel-coronavirus-2019.html (English)
16-Jan-20	● The first known case of COVID-19 in Japan is diagnosed in Kanagawa (Neighboring prefecture of Tokyo)		https://www.mhlw.go.jp/stf/newpage_08906.html (Japanese)
23-Jan-20	○ Chinese government imposes a lockdown in Wuhan.		https://doi.org/10.1016/S1473-3099(21)00166-3 (English)
		1st case of COVID-19 in Japan confirmed by TMG<br>1st Tokyo Metropolitan Emergency Management Council	https://translation2.j-server.com/LUCAITBSAI/ns/tl.cgi/https:/www.bousai.metro.tokyo.lg.jp/taisaku/saigai/1007288/1007293.html?SLANG=ja&TLANG=en&XMODE=0&XCHARSET=utf-8&XJSID=0 (English)

© 2023 Imamura T et al. *JAMA Network Open.*

28-Jan-20	● COVID-19 is classified as a reportable infectious disease under the Infectious Diseases Control Law		https://www.mhlw.go.jp/content/10900000/000589747.pdf (Japanese)
29-Jan-20	● The first charter flight from Wuhan lands at Haneda Airport, Tokyo		https://www.mhlw.go.jp/stf/newpage_09199.html (Japanese)
30-Jan-20	○ WHO declares the outbreak a Public Health Emergency of International Concern (PHEIC)		https://www.who.int/publications/m/item/covid-19-public-health-emergency-of-international-concern-(pheic)-global-research-and-innovation-forum (English)
30-Jan-20	● Japanese government forms Novel Coronavirus Response Headquarters		https://www.kantei.go.jp/jp/singi/novel_coronavirus/kanjikai/sidai_r020130.pdf (Japanese)
03-Feb-20	● A cruise ship named the Diamond Princess arrives in Yokohama, Kanagawa (Neighboring prefecture to Tokyo)		https://www.niid.go.jp/niid/en/2019-ncov-e/9407-covid-dp-fe-01.html (English)
11-Feb-20	○WHO names the new coronavirus "COVID-19"		https://www.who.int/docs/default-source/coronaviruse/transcripts/who-audio-emergencies-coronavirus-full-press-conference-11feb2020-final.pdf (English)
16-Feb-20		▲Announcement of a COVID-19 outbreak resulting from a New Year's Party held on a party boat	https://www.metro.tokyo.lg.jp/tosei/hodohappyo/press/2020/02/13/23.html (Japanese) https://www.metro.tokyo.lg.jp/tosei/hodohappyo/press/2020/02/14/31.html (Japanese) https://www.metro.tokyo.lg.jp/tosei/hodohappyo/press/2020/02/16/01.html (Japanese)
17-Feb-20	● MHLW published A guide to seeking public consultation and medical care		https://www.mhlw.go.jp/content/000603937.pdf (Japanese)

© 2023 Imamura T et al. *JAMA Network Open.*

25-Feb-20	● MHLW forms COVID-19 Cluster Taskforce		https://www.mhlw.go.jp/stf/newpage_09743.html (Japanese)
27-Feb-20	● Prime minister requests school closures for primary and junior high schools		https://www.kantei.go.jp/jp/98_abe/actions/202002/27corona.html (Prime Minister's Office) (Japanese) https://www.mext.go.jp/content/202002228-mxt_kouhou01-000004520_1.pdf (Ministry of education) (Japanese)
06-Mar-20	● National medical insurance coverage is established for COVID-19 PCR testing	Financial support to encourage remote work for companies in Tokyo	https://www.mhlw.go.jp/content/000604470.pdf (Japanese) https://www.metro.tokyo.lg.jp/tosei/hodohappyo/press/2020/03/05/27.html (Japanese)
11-Mar-20	○WHO declares COVID-19 a pandemic		https://pubmed.ncbi.nlm.nih.gov/32191675/ (English)
18-Mar-20	● COVID-19 Cluster Taskforce started technical support at the Tokyo metropolitan government		https://www.metro.tokyo.lg.jp/tosei/governor/governor/kishakaiken/2020/03/documents/20200323_06.pdf (Japanese) https://www.fukushihoken.metro.tokyo.lg.jp/iryo/kansen/corona_portal/link/genzyoubunnseki.html (Japanese)
23-Mar-20		Tokyo Governor requests citizens to avoid 3C's	https://www.metro.tokyo.lg.jp/tosei/governor/governor/kishakaiken/2020/03/23.html (Japanese)
25-Mar-20	● Tokyo 2020 Olympics officially postponed		https://www.2020games.metro.tokyo.lg.jp/news/2020/0325_3198.html (Japanese)
30-Mar-20		▲Tokyo Governor requests the public to avoid visiting nightlife establishments	https://www.metro.tokyo.lg.jp/tosei/governor/governor/kishakaiken/2020/03/30.html (Japanese)
31-Mar-20	● MOFA raised the warning on Infectious Disease to Level 3 (avoiding all travel) in 73		https://www.mofa.go.jp/press/kaiken/kaiken4e_000775.html (English)

	countries and regions		
07-Apr-20	● Declaration of a State of Emergency in seven prefectures		https://japan.kantei.go.jp/ongoingtopics/_00018.html (English)
08-Apr-20	○ Chinese government lifted the Wuhan lockdown		https://www.cn.emb-japan.go.jp/files/100042327.pdf (Japanese) https://www.hubei.gov.cn/zhuanti/2020/gzxxgzbd/zxtb/202004/t20200407_2207131.shtml (Chinese)
10-Apr-20		▲TMG requests business closures for nightlife establishments, schools and universities, gyms, theaters, recreational facilities, and business offices.<br>TMG also requests restaurants limit business hours from 5 am to 8 pm, excepting takeout and deliveries.<br>(from April 11th to May 25th)	https://translation2.j-server.com/LUCAITBSAI/ns/tl.cgi/https:/www.bousai.metro.tokyo.lg.jp/_res/projects/default_project/_page_/001/007/661/2020041000.pdf?SLANG=ja&TLANG=en&XMODE=0&XPARAM=q,&XCHARSET=UTF-8&XPORG=,&XJSID=0 (English)
11-Apr-20		Tokyo Governor announces measures to strengthen healthcare capacity, including increased PCR testing and promoting stay-at-home measures.	https://www.bousai.metro.tokyo.lg.jp/taisaku/saigai/1010035/1015695/1018228/1018247.html (Japanese)
16-Apr-20	● State of Emergency extended to all prefectures		https://japan.kantei.go.jp/ongoingtopics/_00020.html (English)
22-Apr-20	●Expert meeting recommends 80% human mobility reduction, limiting unnecessary trips out of the home, implementing telework, etc.		https://corona.go.jp/expert-meeting/pdf/senmonka_sidai_r020422.pdf (Japanese)

25-Apr-20		TMG promoting stay-at-home measures (from April 25th to May 6th)	https://www.metro.tokyo.lg.jp/tosei/governor/governor/kishakaiken/2020/04/documents/20200043_01.pdf (Japanese)
14-May-20	● State of Emergency lifted except for 8 prefectures, including Tokyo		https://corona.go.jp/news/pdf/kinkyujitaisengen_gaiyou0514.pdf (Japanese)
22-May-20		▲TMG announces a roadmap to prepare for the next epidemic	https://www.metro.tokyo.lg.jp/tosei/hodohappyo/press/2020/05/22/documents/11_00_1.pdf (Japanese)https://www.metro.tokyo.lg.jp/tosei/hodohappyo/press/2020/05/22/11.html (Japanese)https://www.metro.tokyo.lg.jp/tosei/hodohappyo/press/2020/05/22/documents/11_01_1.pdf (Japanese)
25-May-20	● State of Emergency lifted for the remaining 8 prefectures, including Tokyo	26th Tokyo Metropolitan Government COVID-19 Response Headquarters Meeting	https://www.bousai.metro.tokyo.lg.jp/taisaku/saigai/1007288/1008114.html (Japanese)
26-May-20		▲Transition to Step 1 of the TMG roadmap, a schedule of stepwise relaxation of restrictions and infection control measures<br>(from May 26th to May 31st)	https://www.bousai.metro.tokyo.lg.jp/taisaku/saigai/1007288/1008114.html (Japanese)
01-Jun-20		▲Transition to Step 2 of the TMG roadmap, relaxing restrictions<br>(from June 1st to June 11th)	https://www.bousai.metro.tokyo.lg.jp/_res/projects/default_project/_page_/001/008/166/20200052904.pdf (Japanese)
02-Jun-20		▲TMG declares the “Tokyo Alert,” requesting adherence to infection prevention measures and behaviors, including	https://www.bousai.metro.tokyo.lg.jp/_res/projects/default_project/_page_/001/008/242/20200603.pdf (Japanese)

© 2023 Imamura T et al. *JAMA Network Open.*

		avoiding nightlife establishments and promoting remote work (from June 2nd to June 11th)	
11-Jun-20		▲TMG lifts the "Tokyo Alert"	https://www.bousai.metro.tokyo.lg.jp/_res/projects/default_project/_page_/001/009/652/20200611n.pdf (Japanese)
12-Jun-20		▲Transition to Step 3 of the TMG roadmap, relaxing restrictions (from June 12th to June 18th)	https://www.bousai.metro.tokyo.lg.jp/taisaku/saigai/1007288/1008462.html (Japanese)<br>https://www.bousai.metro.tokyo.lg.jp/_res/projects/default_project/_page_/001/008/462/2020061113.pdf (Japanese)<br>https://www.koho.metro.tokyo.lg.jp/diary/report/2020/06/02/15/01.html (Japanese)
19-Jun-20	● Relaxation of the request for inter-prefectural travel<br>Professional baseball games start (without spectators)<br>Relaxation of events restrictions		https://www.kantei.go.jp/jp/98_abe/actions/202006/18corona.html (Japanese)
24-Jun-20	● Dissolution of the Expert meeting on COVID-19		https://www3.nhk.or.jp/news/html/20200624/k10012482591000.html (Japanese)<br>https://www.kantei.go.jp/jp/singi/novel_coronavirus/th_siryou/sidai_r020703.pdf (Japanese)
02-Jul-20		▲Tokyo Governor warns of the resurgence of COVID-19 cases and advises against visiting nightlife establishments, especially in Shinjuku and Ikebukuro	https://www.bousai.metro.tokyo.lg.jp/taisaku/saigai/1007288/1009649.html (Japanese)<br>https://www.koho.metro.tokyo.lg.jp/diary/report/2020/07/03/01.html (Japanese)

© 2023 Imamura T et al. *JAMA Network Open.*

03-Jul-20	● Establishment of the COVID-19 subcommittee		
15-Jul-20		Tokyo Governor warning that COVID-19 epidemic in Tokyo is at the most severe stage	https://www.bousai.metro.tokyo.lg.jp/taisaku/saigai/1007288/1009753.html (Japanese)https://www.bousai.metro.tokyo.lg.jp/_res/projects/default_project/_page_/001/009/753/gijiroku/gijiroku20200715.pdf (Japanese)https://www.koho.metro.tokyo.lg.jp/diary/report/2020/07/17/01.html (Japanese)
20-Jul-20		TMG promoting remote work	https://www.metro.tokyo.lg.jp/tosei/hodohappyo/press/2020/06/12/07.html (Japanese)
22-Jul-20	● "Go to Travel" campaign started all over Japan, excluding Tokyo		https://www.mlit.go.jp/report/interview/daijin200717.html (Japanese)
03-Aug-20		▲TMG requests restaurants and bars serving alcohol and karaoke establishments in Tokyo to limit their business hours between 5 am and 10 pm.<br>(from August 3rd to August 31st)	https://www.bousai.metro.tokyo.lg.jp/taisaku/saigai/1007288/1009999.html (Japanese) https://www.bousai.metro.tokyo.lg.jp/_res/projects/default_project/_page_/001/009/999/35kai/gijiroku20200730.pdf.pdf (Japanese) https://www.koho.metro.tokyo.lg.jp/diary/report/2020/07/30/02.html (Japanese)
28-Aug-20	● The government announces a new strategy regarding the healthcare system, testing capacity, and vaccine roll-out		https://www.kantei.go.jp/jp/singi/novel_coronavirus/th_siryou/sidai_r020828.pdf (Japanese)
01-Sep-20		▲TMG continues to request restaurants, bars, and karaoke establishments operating in central TMG to limit their business hours between 5 am and 10 pm	https://www.bousai.metro.tokyo.lg.jp/taisaku/saigai/1007288/1011491.html (Japanese) https://www.bousai.metro.tokyo.lg.jp/_res/projects/default_project/_page_/001/011/491/gijiroku/gijiroku_20200827

© 2023 Imamura T et al. *JAMA Network Open.*

		(from September 1st to September 15th)	.pdf (Japanese) https://www.koho.metro.tokyo.lg.jp/diary/report/2020/08/27/01.html (Japanese)
17-Sep-20	● Inauguration of prime minister Suga and his new cabinet		https://www.kantei.go.jp/jp/99_suga/statement/2020/0916kaiken.html (Japanese)
01-Oct-20	● "Go to Eat" campaign started all over Japan<br>● "Go to Travel" campaign in Tokyo s commences	TMG establishes Tokyo Center for Infectious Disease Control and Prevention	https://www.metro.tokyo.lg.jp/tosei/hodohappyo/press/2020/09/25/12.html (Japanese)
23-Oct-20		▲TMG's "Motto Tokyo" campaign promotes travels inside Tokyo	https://motto-tokyo.jp (Japanese)
19-Nov-20		Relaxation of the size limits for events and gatherings (from December 1st, 2020, to February 28th, 2021)	https://www.bousai.metro.tokyo.lg.jp/_res/projects/default_project/_page_/001/012/264/20201111904.pdf (Japanese)
26-Nov-20		▲TMG requests voluntary closure of restaurants and bars and limiting hours for other businesses	https://www.bousai.metro.tokyo.lg.jp/taisaku/saigai/1007288/1012312.html (Japanese) https://www.metro.tokyo.lg.jp/tosei/governor/governor/kishakaiken/2020/11/25.html (Japanese)
27-Nov-20		▲TMG announces cessation of the "Go to Eat" campaign in Tokyo	https://www.bousai.metro.tokyo.lg.jp/taisaku/saigai/1007288/1012312.html (Japanese) https://www.metro.tokyo.lg.jp/tosei/governor/governor/kishakaiken/2020/11/25.html (Japanese) https://www.bousai.metro.tokyo.lg.jp/_res/projects/default_project/_page_/001/012/455/20201121403.pdf (Japanese)
28-Nov-20		▲TMG ceases "Motto Tokyo" campaign	https://www.bousai.metro.tokyo.lg.jp/taisaku/saigai/1007288/1012312.html (Japanese) https://www.metro.toky

© 2023 Imamura T et al. *JAMA Network Open.*

			o.lg.jp/tosei/governor/governor/kishakaiken/2020/11/25.html (Japanese)
		▲TMG continues to request restaurants, bars, and karaoke establishments to limit their business hours between 5 am and 10 pm.<br>(from November 28th, 2020, to January 11th, 2021)	https://www.bousai.metro.tokyo.lg.jp/taisaku/saigai/1007288/1012312.html (Japanese)
01-Dec-20		TMG announces guidelines for limiting the sizes of events and social gatherings<br>(from December 1st, 2020, to February 28th, 2021)	https://www.bousai.metro.tokyo.lg.jp/_res/projects/default_project/_page_/001/009/761/1/13_.pdf (Japanese)
08-Dec-20		Governors of Tokyo and three other prefectures in metropolitan areas announce a joint message regarding infection prevention	https://www.seisakukikaku.metro.tokyo.lg.jp/collaboration/covid19/20201208message.html (Japanese)
16-Dec-20		TMG activates a health care facility dedicated to COVID-19	https://www.byouin.metro.tokyo.lg.jp/about/houdou/pdf/f4dae21db9ca6a8e794299373b17e783d526c0cd.pdf (Japanese)
16-Dec-20		Governors of Tokyo and three other prefectures request the cancellation of all-night public transportation services on New Year's Eve	https://www.metro.tokyo.lg.jp/tosei/hodohappyo/press/2020/12/18/01.html (Japanese)
17-Dec-20	● Ministry of Land, Infrastructure, Transport, and Tourism requests the cancellation of all-night public transportation on New Year's Eve in Tokyo and three other		https://www.mlit.go.jp/report/interview/daijin201218.html (Japanese)

© 2023 Imamura T et al. *JAMA Network Open.*

	prefectures in the metropolitan area		
18-Dec-20		TMG advises against winter holiday lighting events	https://www.toshiseibi.metro.tokyo.lg.jp/seisaku/fop_town/pdf/illumi_02.pdf (Japanese)
23-Dec-20	● COVID-19 Cluster Taskforce ends technical support at the TMG	TMG closes public gyms during the end-of-year and new-year period (from December 23rd, 2020, to January 11th, 2021)	https://www.metro.tokyo.lg.jp/tosei/hodohappyo/press/2020/12/23/21.html (Japanese)
28-Dec-20	● "Go to Travel" campaign is paused		https://www.kantei.go.jp/jp/singi/novel_coronavirus/th_siryou/t_gaiyou_r021214.pdf (Japanese)

TMG: Tokyo Metropolitan Government
MHLW: Ministry of Health, Labour and Welfare of Japan
MOFA: Ministry of Foreign Affairs of Japan
"Go to Travel" campaign: governmental schemes to promote internal travel
"Go to Eat" campaign: governmental schemes to encourage people to dine out
Motto Tokyo campaign: TMG scheme targeting all residents in Tokyo to promote travel within Tokyo

○: International event
●: domestic event
▲: Measures by TMG related to nightlife establishments

[References]

1. Yoshikawa E, Fukumoto M, Iguchi A, et al. Guide on Active Epidemiological Investigation for Public Health Nurses In Response to COVID-19 in Japan (2nd edition). Accessed July 1, 2022. https://plaza.umin.ac.jp/~COVID19/core/survey_guide_for_PHN_EN_2nd%20edition.pdf
2. Imamura T, Saito T, Oshitani H. Roles of Public Health Centers and Cluster-Based Approach for COVID-19 Response in Japan. *Health Secur.* Dec 18 2020;19(2):229-31. doi:10.1089/hs.2020.0159
3. Infectious Disease Surveillance Center National Institute of Infectious Diseases. Infectious Disease Surveillance System in Japan. Accessed June 21, 2022. https://www.niid.go.jp/niid/images/epi/nesid/nesid_en.pdf
4. Ministry of Health Labour and Welfare. Health Center Real-time Information-sharing System on COVID-19 (HER-SYS). Accessed June 21, 2022. https://www.mhlw.go.jp/content/10900000/000678061.pdf
5. Ministry of Health Labour and Welfare. Diagnostic tests of COVID-19. Accessed June 21, 2022. https://www.mhlw.go.jp/stf/seisakunitsuite/bunya/0000121431_00132.html

© 2023 Imamura T et al. *JAMA Network Open.*

6. Tokumoto A, Akaba H, Oshitani H, et al. COVID-19 health system response monitor: Japan. World Health Organization Regional Office for South-East Asia. Accessed July 1, 2022. https://apps.who.int/iris/bitstream/handle/10665/338399/9789290228264-eng.pdf

© 2023 Imamura T et al. *JAMA Network Open.*

eFigure 1. Concept of COVID-19 Transmission Settings and Onward Transmission

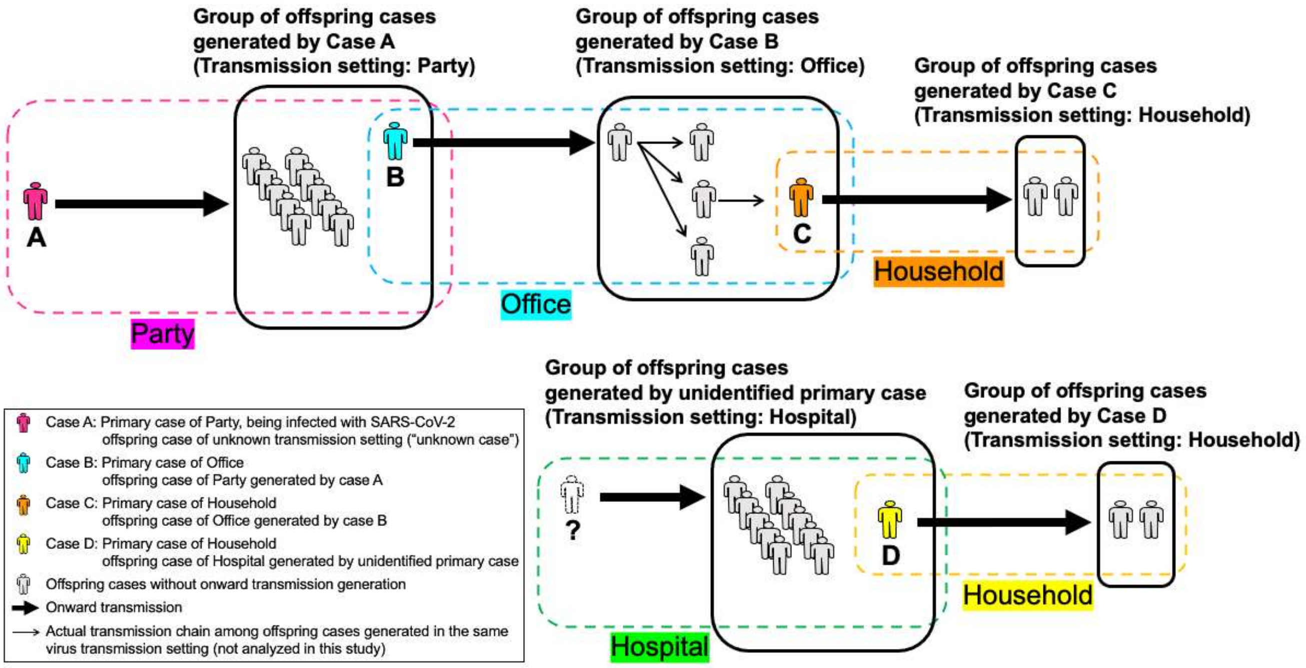

We defined a transmission setting as an occasion where the epidemiological link was identified, such as party, office, hospital, or household settings. One epidemiological link can involve multiple transmission settings; for example, if a party event results in transmission at an office and then at a household, cases in the same link were assigned to three different transmission settings: party, office, and household settings. A primary case was identified in each transmission setting: case A in a party, case B in an office, and cases C and D in respective households. The rest of the cases in transmission settings were defined as offspring cases. We did not identify primary cases in health care settings. Onward transmission is a transmission from one setting to another, shown in bold arrows. This study did not analyze multiple generations of transmission among offspring cases involved in the same transmission settings and onward transmission towards health care settings.

© 2023 Imamura T et al. *JAMA Network Open.*

eTable 1. Definition of Cases According to Their Identified Transmission Settings

Transmission setting	Definition
Imported	A case was defined as one likely to be infected at imported setting when the case had a travel history to countries or areas with COVID-19 cases reported to WHO within 14 days prior to symptom onset or 14 days prior to diagnosis when the case is asymptomatic.
Nightlife	A case was defined as one likely to be infected at nightlife setting when the case was presumed to be infected with SARS-CoV-2 at restaurants, bars, pubs, nightclubs (including host and hostess clubs), and other eating or drinking establishments at nighttime. Both clients and employees of these establishments were included in nightlife cases. Cases identified at nightlife setting were further classified into those associated with and without hosts and hostesses. Nightlife establishments with hosts and hostesses are unique and popular in Japan, which cater to female customers (host clubs) and male customers (hostess clubs)[1,2]. Establishments offering sexual services were also classified into the nightlife establishments with hosts and hostesses.
Dining	A case was defined as one likely to be infected at dining setting when the case was presumed to be infected with SARS-CoV-2 on occasions including eating/drinking but did not fulfill the definition of cases identified at nightlife setting. The dining settings included home parties, restaurants and bars during daytime or outdoor barbeques and picnics.
Workplace	A case was defined as one likely to be infected at workplace setting when the case was presumed to be infected with SARS-CoV-2 at work-related environments such as office and business meetings. In case the transmission event involved eating/drinking, such as dinner with business partners, the event was classified either into nightlife or dining setting.
Household	A case was defined as one likely to be infected at household setting when the case was presumed to be infected with SARS-CoV-2 at the household. A household case also includes those likely to be infected from a confirmed COVID-19 case within his or her second degree of kinship, regardless of living in the same household or not.
Health care	A case was defined as one likely to be infected at health care setting when the case was presumed to be infected with SARS-COV-2 at health care facilities, including hospitals, outpatient facilities, long-term care facilities for the elderly and disabled, and day-care centers. Health care cases included patients or residents of the facilities, staff, visitors, and others. We did not determine a primary case who transmitted SARS-CoV-2 into health care setting since identifying a possible primary case in a health care setting was difficult even with a detailed epidemiological investigation.
Other	A case was identified as one likely to be infected at other setting when the case was presumed to be infected with SARS-CoV-2 in a known transmission setting other than the previously defined six settings. Other settings included theater and music-related events, gymnasium and sports-related events, universities, nurseries, schools and other types of children-related facilities, and others.
Unknown	A case was defined as one with unknown transmission setting when the case was not classified into either of the seven settings above. Unknown cases were further classified into those with or without documented history of visiting nightlife establishments.

Cases that tested positive for COVID-19 at border control were not included in this study. All the imported cases in this study tested negative upon entry to Japan but were later confirmed as COVID-19 cases.

[References]
1. Kamise Y. Occupational stigma and coping strategies of women engaged in the commercial sex industry: A study on the perception of “Kyaba-Cula hostesses” in Japan. *Sex Roles.* 2013; 69: 42–57.
2. Takeyama A. Beauty of seduction in a Tokyo host club. In Hymans TD, Kershof N (Ed.), *International Institute for Asian Studies.* 2006: 9. retrieved from https://www.iias.asia/sites/iias/files/theNewsletter/2019-06/IIAS_NL40_FULL.pdf

© 2023 Imamura T et al. *JAMA Network Open.*

eTable 2. Characteristics of 44 054 COVID-19 Cases by Waves in Tokyo Between January and December 2020

Characteristics	Cases, No. (%) Total	Wave1 (Jan. 1st-May 25th, 2020)	Wave2 (May 26th-Sep. 30th, 2020)	Wave3 (Oct. 1st-Dec. 6th, 2020)
Cases, No.	44054	5173	20858	18023
Sex				
Male	25241 (57.3)	2954 (57.1)	12191 (58.4)	10096 (56.0) [a]
Female	18813 (42.7)	2219 (42.9)	8667 (41.6)	7927 (44.0)
Age, y				
Median (IQR)	36 (26-52)	47 (32-64)	33 (25-47)	38 (27-54) [a]
0-17	1802 (4.1)	119 (2.3)	772 (3.7)	911 (5.1) [a]
18-39	22833 (51.8)	1863 (36.0)	12562 (60.2)	8408 (46.7)
40-64	13749 (31.2)	1924 (37.2)	5662 (27.1)	6163 (34.2)
≥65	5670 (12.9)	1267 (24.5)	1862 (8.9)	2541 (14.1)
Symptom				
Asymptomatic	6904 (15.7)	371 (7.2)	2581 (12.4)	3952 (21.9) [a]
Symptomatic without known date of onset	6454 (14.7)	477 (9.2)	3254 (15.6)	2723 (15.1)
Symptomatic with known date of onset	30696 (69.7)	4325 (83.6)	15023 (72.0)	11348 (63.0)
Time from onset to confirmation, median (IQR), d				
Overall	4 (2-7)	8 (5-10)	4 (3-6)	3 (2-5) [a]
Outcome				
Died	563 (1.3)	332 (6.4)	127 (0.6)	104 (0.6) [a]
Survived	43491 (98.7)	4841 (93.6)	20731 (99.4)	17919 (99.4)
Transmission setting				
Imported	152 (0.3)	123 (2.4)	8 (0.0)	21 (0.1) [a]
Nightlife	1174 (2.7)	122 (2.4)	900 (4.3)	152 (0.8)
Dining	275 (0.6)	13 (0.3)	169 (0.8)	93 (0.5)
Workplace	702 (1.6)	64 (1.2)	324 (1.6)	314 (1.7)
Household	6768 (15.4)	683 (13.2)	2832 (13.6)	3253 (18.0)
Health care	2733 (6.2)	1069 (20.7)	697 (3.3)	967 (5.4)
Other	1318 (3.0)	58 (1.1)	570 (2.7)	690 (3.8)
Unknown	30932 (70.2)	3041 (58.8)	15358 (73.6)	12533 (69.5)

[a] $P < .001$. The Kruskal-Wallis and the $\chi 2$ tests were utilized to evaluate differences between the three waves regarding continuous and categorical variables, respectively.

© 2023 Imamura T et al. *JAMA Network Open.*

eTable 3. Association Between COVID-19–Related Death and Characteristics of Cases

Characteristics	Survived, No.	Died, No. (CFR(%))	OR (95%CI)[a]	*P* value	aOR (95%CI)[b]	*P* value
Cases	43491	563 (1.28)				
Sex						
Female	18603	210 (1.12)	1 [Reference]	NA	1 [Reference]	NA
Male	24888	353 (1.40)	1.26 (1.06-1.49)	0.009	1.90 (1.57-2.31)	<0.001
Age group, y						
<Group 1>						
0-17	1802	0 (0.00)	NA			
18-39	22830	3 (0.01)	1 [Reference]	NA		
40-64	13693	56 (0.41)	31.12 (9.74-99.45)	<0.001		
≥65	5166	504 (8.89)	742.44 (238.55-2310.67)	<0.001		
<Group 2>						
<65	38325	59 (0.15)	1 [Reference]	NA	1 [Reference]	NA
≥65	5166	504 (8.89)	63.37 (48.32-83.12)	<0.001	39.85 (30.05-52.85)	<0.001
Transmission setting						
<Classification 1>						
Imported	149	3 (1.97)	2.33 (0.72-7.52)	0.16		
Nightlife	1171	3 (0.26)	0.30 (0.09-0.95)	0.04		
Dining	275	0 (0.00)	NA			
Workplace	700	2 (0.28)	0.33 (0.08-1.36)	0.12		
Household	6710	58 (0.86)	1 [Reference]	NA		
Health care	2465	268 (9.81)	12.58 (9.43-16.77)	<0.001		
Other	1315	3 (0.23)	0.26 (0.08-0.84)	0.03		
Unknown	30706	226 (0.73)	0.85 (0.64-1.14)	0.28		
<Classification 2>						
Household	6710	58 (0.86)	1 [Reference]	NA	1 [Reference]	NA
Hospital/welfare	2465	268 (9.81)	12.58 (9.43-16.77)	<0.001	3.32 (2.45-4.51)	<0.001
Others[c]	3610	11 (0.30)	0.35 (0.18-0.67)	0.002	0.64 (0.33-1.26)	0.20
Unknown	30706	226 (0.73)	0.85 (0.64-1.14)	0.28	1.16 (0.86-1.58)	0.33
Time from onset to confirmation, d						
≤3	12674	167 (1.30)	1 [Reference]	NA		
≥4	17587	268 (1.50)	1.16 (0.95-1.40)	0.14		
No Data[d]	13230	128 (0.96)	0.73 (0.58-0.93)	0.009		
Wave						
1	4841	332 (6.42)	1 [Reference]	NA	1 [Reference]	NA
2	20731	127 (0.61)	0.09 (0.07-0.11)	<0.001	0.23 (0.18-0.29)	<0.001
3	17919	104 (0.58)	0.08 (0.07-0.11)	<0.001	0.14 (0.11-0.18)	<0.001

Abbreviations: aOR, adjusted odds ratio; CFR, case fatality ratio; NA, not applicable; OR, odds ratio

[a] Factors associated with death were compared using logistic regression.

[b] In the multivariable analysis, the odds ratio for the fatal outcome was adjusted for sex, age group (<65 years, ≥65 years), transmission settings (household, health care, unknown, and all the other settings including imported, nightlife, dining, workplace, and other), and wave. Due to the small number of fatal cases, cases aged younger than 65 years old and cases involved in settings other than household, health care, or unknown were grouped as appropriate.

[c] Cases identified in transmission settings other than household, health care, or unknown settings.

[d] Including both asymptomatic cases and symptomatic cases without known days of onset

© 2023 Imamura T et al. *JAMA Network Open.*

eTable 4. Number of Transmission Settings and Characteristics of their Primary Cases by the Number of Offspring Cases per Setting

	Total, No.	Offspring cases per setting			
		1 case, No. (%)	2-4 cases, No. (%)	≥ 5 cases, No. (%)	
Trasnmission settings					
Settings	6624	4609 (69.6)	1720 (26.0)	295 (4.5)	
Categories[a]					
Nightlife	380	200 (52.6)	108 (28.4)	72 (18.9)	[Reference]
Dining	205	164 (80.0)	37 (18.0)	4 (2.0)	***
Workplace	285	192 (67.4)	72 (25.3)	21 (7.4)	***
Household	4886	3563 (72.9)	1292 (26.4)	31 (0.6)	***
Health care	329	102 (31.0)	108 (32.8)	119 (36.2)	***
Other	539	388 (72.0)	103 (19.1)	48 (8.9)	***
Primary cases[b]					
Cases	6295	4507 (71.6)	1612 (25.6)	176 (2.8)	
Registeration in Tokyo					
Unregistered[c]	174	154 (88.5)	20 (11.5)	0 (0.0)	*
Registered[d]	6121	4353 (71.1)	1592 (26.0)	176 (2.9)	
Imported	18	10 (55.6)	6 (33.3)	2 (11.1)	
Nightlife	109	82 (75.2)	26 (23.9)	1 (0.9)	
Dining	25	19 (76.0)	5 (20.0)	1 (4.0)	
Workplace	79	52 (65.8)	27 (34.2)	0 (0.0)	
Household	37	32 (86.5)	5 (13.5)	0 (0.0)	
Health care	170	127 (74.7)	42 (24.7)	1 (0.6)	
Other	154	111 (72.1)	39 (25.3)	4 (2.6)	
Unknown	5529	3920 (70.9)	1442 (26.1)	167 (3.0)	
History of nightlife visits[e]					
With history	712	484 (68.0)	156 (21.9)	72 (10.1)	***
Without history	4817	3436 (71.3)	1286 (26.7)	95 (2.0)	
Sex					
Female	2343	1646 (70.3)	638 (27.2)	59 (2.5)	
Male	3778	2707 (71.7)	954 (25.3)	117 (3.1)	
Age group[f], y					
0-17	210	111 (52.9)	89 (42.4)	10 (4.8)	
18-39	2686	1921 (71.5)	640 (23.8)	125 (4.7)	[Reference]
40-64	2307	1618 (70.1)	661 (28.7)	28 (1.2)	***
≥65	918	703 (76.6)	202 (22.0)	13 (1.4)	***
Symptoms					
Asymptomatic	406	299 (73.6)	98 (24.1)	9 (2.2)	
Symptomatic	5715	4054 (70.9)	1494 (26.1)	167 (2.9)	
Time from onset to confirmation[g], d					
≤3	1813	1298 (71.6)	473 (26.1)	42 (2.3)	[Reference]
≥4	3079	2159 (70.1)	798 (25.9)	122 (4.0)	**
No Data[h]	1229	896 (72.9)	321 (26.1)	12 (1.0)	
Wave					
1	586	418 (71.3)	150 (25.6)	18 (3.1)	*
2	2851	2026 (71.1)	726 (25.5)	99 (3.5)	
3	2684	1909 (71.1)	716 (26.7)	59 (2.2)	
Outcome					
Survived	6020	4284 (71.2)	1564 (26.0)	172 (2.9)	
Died	101	69 (68.3)	28 (27.7)	4 (4.0)	

[a] Proportion of settings with offspring cases 1-4 / ≥ 5 were compared between nightlife setting and other settings using the $\chi 2$ test with Šidák corrections

[b] The same primary case could be counted multiple times if he/she was a primary case of two or

© 2023 Imamura T et al. *JAMA Network Open.*

more settings. Primary cases of healthcare settings were not identified in this study. Proportion of primary cases with offspring cases 1-4 / ≥ 5 were compared between different categories of primary cases using the $\chi 2$ test.

[c] Detailed epidemiological data was not available due to the registration outside of Tokyo or incomplete registration during our study period in Tokyo

[d] Primary cases registered in Tokyo during our study period

[e] Having a history of visiting nightlife establishments regarding Unknown cases

[f] Proportion of primary cases with offspring cases 1-4 / ≥ 5 were compared between 18-39 years and other age groups using the $\chi 2$ test with Šidák corrections

[g] Proportion of primary cases with offspring cases 1-4 / ≥ 5 were compared between cases with onset-to-confirmation of ≤3 days and cases with ≥4 days or without data using the $\chi 2$ test with Šidák corrections

[h] Including both asymptomatic cases and symptomatic cases without known days of onset

* *P* < .05
** *P* < .01
*** *P* < .001

© 2023 Imamura T et al. *JAMA Network Open.*

eTable 5. Association of Transmission Settings and Other Factors With the Generation of Total Onward Transmission, to Nonhousehold Settings, and to Household Settings: Univariable Analysis[a]

Characteristics	Total No. (%) Without OWT[b]	With OWT[c]	OR (95%CI)	*P* value	OWT Towards non-household No. (%) Without OWT[b]	With OWT[c]	OR (95%CI)	*P* value	Towards household No. (%) Without OWT[d]	With OWT[e]	OR (95%CI)	*P* value
Cases	12540 (95.56)	582 (4.44)	NA	NA	13034 (99.33)	88 (0.67)	NA	NA	5854 (92.13)	500 (7.87)	NA	NA
Transmission setting												
Imported	134 (88.16)	18 (11.84)	1.30 (0.76-2.23)	0.34	150 (98.68)	2 (1.32)	0.59 (0.14-2.51)	0.48	136 (89.47)	16 (10.53)	1.53 (0.86-2.72)	0.15
Nightlife	1068 (90.97)	106 (9.03)	ref.		1148 (97.79)	26 (2.21)	ref.		1093 (93.10)	81 (6.90)	ref.	
Dining	252 (91.64)	23 (8.36)	0.89 (0.55-1.44)	0.63	270 (98.18)	5 (1.82)	0.82 (0.31-2.15)	0.69	255 (92.73)	20 (7.27)	1.03 (0.61-1.73)	0.93
Workplace	624 (88.89)	78 (11.11)	1.20 (0.84-1.71)	0.31	699 (99.57)	3 (0.43)	0.19 (0.06-0.62)	0.006	626 (89.17)	76 (10.83)	1.58 (1.09-2.30)	0.02
Household	6732 (99.47)	36 (0.53)	0.05 (0.03-0.08)	<0.001	6732 (99.47)	36 (0.53)	0.24 (0.14-0.39)	<0.001	NA	NA		
Health care	2563 (93.78)	170 (6.22)	0.78 (0.58-1.05)	0.10	2732 (99.96)	1 (0.04)	0.02 (0.00-0.11)	<0.001	2564 (93.82)	169 (6.18)	1.04 (0.76-1.43)	0.81
Other	1167 (88.54)	151 (11.46)	1.30 (0.97-1.73)	0.08	1303 (98.86)	15 (1.14)	0.50 (0.27-0.96)	0.04	1180 (89.53)	138 (10.47)	1.55 (1.13-2.13)	0.007
Sex												
Female	6754 (95.98)	283 (4.02)	1 [Reference]	NA	6993 (99.37)	44 (0.63)	1 [Reference]	NA	2781 (91.99)	242 (8.01)	1 [Reference]	NA
Male	5786 (95.09)	299 (4.91)	1.28 (1.07-1.53)	0.007	6041 (99.28)	44 (0.72)	1.15 (0.76-1.75)	0.51	3073 (92.25)	258 (7.75)	0.98 (0.82-1.18)	0.86
Age group, y												
0-17	1264 (95.18)	64 (4.82)	1.11 (0.81-1.53)	0.51	1318 (99.25)	10 (0.75)	0.87 (0.44-1.74)	0.70	145 (72.86)	54 (27.14)	5.21 (3.56-7.62)	<0.001
18-39	5200 (96.05)	214 (3.95)	1 [Reference]	NA	5368 (99.15)	46 (0.85)	1 [Reference]	NA	2999 (94.64)	170 (5.36)	1 [Reference]	NA
40-64	3483 (95.48)	165 (4.52)	1.11 (0.89-1.38)	0.37	3623 (99.31)	25 (0.69)	0.80 (0.49-1.31)	0.37	1264 (89.84)	143 (10.16)	1.87 (1.49-2.36)	<0.001
≥65	2593 (94.91)	139 (5.09)	1.25 (0.98-1.60)	0.08	2725 (99.74)	7 (0.26)	0.31 (0.14-0.68)	0.003	1446 (91.58)	133 (8.42)	1.78 (1.40-2.26)	<0.001
Symptoms												
Asymptomatic	3551 (97.10)	106 (2.90)	1 [Reference]	NA	3649 (99.78)	8 (0.22)	1 [Reference]	NA	1580 (94.10)	99 (5.90)	1 [Reference]	NA
Symptomatic	8989 (94.97)	476 (5.03)	2.05 (1.61-2.61)	<0.001	9385 (99.15)	80 (0.85)	3.86 (1.88-7.93)	<0.001	4274 (91.42)	401 (8.58)	1.56 (1.24-1.95)	<0.001
Time from onset to confirmation, d												
≤3	3789(95.85)	164 (4.15)	1 [Reference]	NA	3923(99.24)	30 (0.76)	1 [Reference]	NA	1788(92.93)	136 (7.07)	1 [Reference]	NA
≥4	3560(93.73)	238 (6.27)	1.56 (1.26-1.94)	<0.001	3758(98.95)	40 (1.05)	1.39 (0.87-2.23)	0.17	1861(90.25)	201 (9.75)	1.36 (1.09-1.70)	0.006
No Data[f]	5191(96.65)	180 (3.35)	0.72 (0.57-0.92)	0.007	5353(99.66)	18 (0.34)	0.44 (0.25-0.79)	0.006	2205(93.12)	163 (6.88)	0.90 (0.72-1.14)	0.38
Wave												
1	2031 (95.26)	101 (4.74)	1 [Reference]	NA	2124 (99.62)	8 (0.38)	1 [Reference]	NA	1355 (93.51)	94 (6.49)	1 [Reference]	NA
2	5229 (95.07)	271 (4.93)	0.85 (0.64-1.14)	0.28	5449 (99.07)	51 (0.93)	2.39 (1.13-5.06)	0.02	2447 (91.72)	221 (8.28)	0.96 (0.71-1.30)	0.80
3	5280 (96.17)	210 (3.83)	0.57 (0.42-0.78)	<0.001	5461 (99.47)	29 (0.53)	1.35 (0.62-2.97)	0.45	2052 (91.73)	185 (8.27)	0.96 (0.70-1.32)	0.79

Abbreviations: NA, not applicable; OR, odds ratio; OWT, onward transmission

[a] Factors associated with the generation of OWT were compared using generalized estimating equations models for accounting within-transmission-setting associations. Nightlife setting, female sex, age 18 to 39 years, asymptomatic cases, and Wave 1 were set as references. Household cases were excluded from the analysis of OWT towards household settings since household cases do not generate OWT towards household settings.

[b] Cases not generating OWT

[c] Cases generating OWT

[d] Cases not generating OWT (excluding household cases)

[e] Cases generating OWT (excluding household cases)

[f] Including both asymptomatic cases and symptomatic cases without known days of onset

© 2023 Imamura T et al. *JAMA Network Open.*

eFigure 2. Comparison of the Interval Between Each Unknown Case's Date of Onset and the Respective Wave's Peak by the History of Visiting Nightlife Establishments

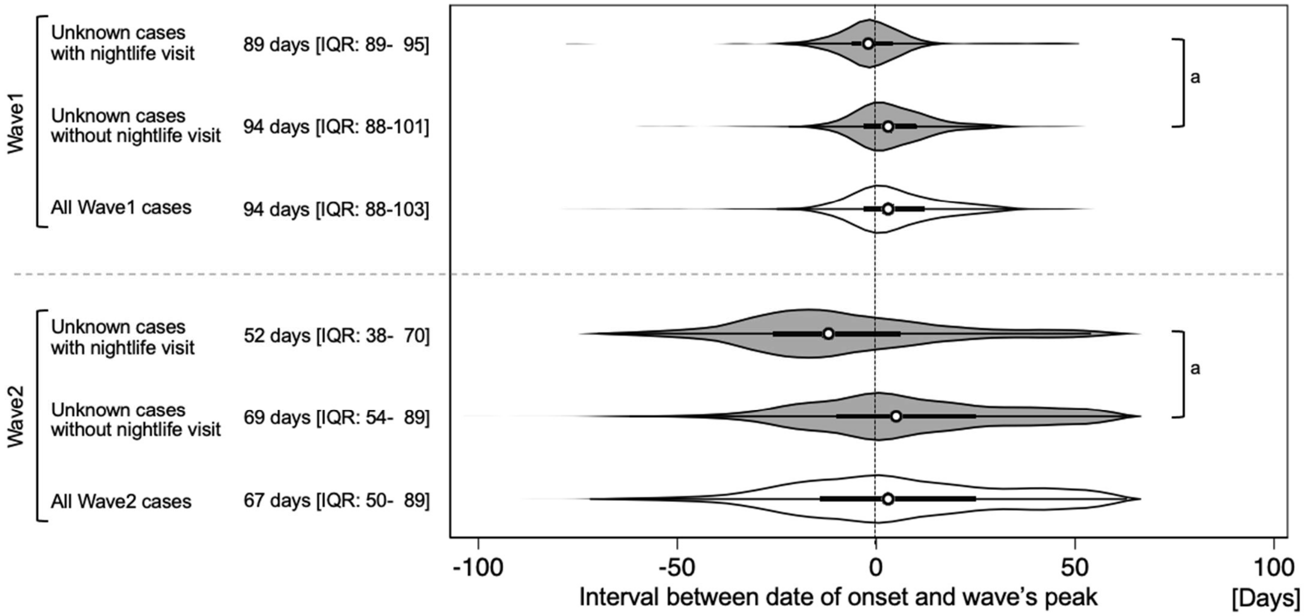

The interval between each unknown case's date of onset and the respective wave's peak (Wave 1: April 1st, 2020; Wave 2: July 29th, 2020) was calculated. The distribution of intervals was plotted regarding unknown cases with a history of visiting nightlife establishments, unknown cases without such history, and all the cases stratified by Wave 1 and Wave 2. The distribution of intervals was compared using the Kruskal-Wallis test with Dunn test since the within-transmission-setting association was not identified among unknown cases.

[a] $P < .001$.

© 2023 Imamura T et al. *JAMA Network Open.*

eTable 6. Association of the History of Visiting Nightlife Establishments and Other Factors With the Generation of Total Onward Transmission, to Nonhousehold Settings, and to Household Settings Among Unknown Cases: A Multivariable Analysis[a]

Characteristics	OWT Total No. (%)				Towards non-household No. (%)				Towards household No. (%)			
	Without OWT[b]	With OWT[c]	aOR (95%CI)	*P* value	Without OWT[b]	With OWT[c]	aOR (95%CI)	*P* value	Without OWT[b]	With OWT[c]	aOR (95%CI)	*P* value
Unknown cases	25609 (82.79)	5323 (17.21)	NA	NA	29749 (96.18)	1183 (3.82)	NA	NA	26625 (86.08)	4307 (13.92)	NA	NA
History of nightlife visits												
Without history	23384 (83.33)	4678 (16.67)	1 [Reference]	NA	27277 (97.20)	785 (2.80)	1 [Reference]	NA	24052 (85.71)	4010 (14.29)	1 [Reference]	NA
With history	2225 (77.53)	645 (22.47)	1.71 (1.56-1.89)	<0.001	2472 (86.13)	398 (13.87)	5.30 (4.64-6.05)	<0.001	2573 (89.65)	297 (10.35)	0.85 (0.75-0.97)	0.01
Sex												
Female	9791 (83.14)	1985 (16.86)	1 [Reference]	NA	11360 (96.47)	416 (3.53)	1 [Reference]	NA	10150 (86.19)	1626 (13.81)	1 [Reference]	NA
Male	15818 (82.57)	3338 (17.43)	0.99 (0.93-1.05)	0.73	18389 (96.00)	767 (4.00)	1.13 (1.00-1.28)	0.05	16475 (86.00)	2681 (14.00)	0.96 (0.89-1.02)	0.19
Age group, y												
0-17	334 (70.46)	140 (29.54)	3.11 (2.53-3.82)	<0.001	454 (95.78)	20 (4.22)	1.47 (0.93-2.32)	0.10	348 (73.42)	126 (26.58)	3.65 (2.95-4.52)	<0.001
18-39	15052 (86.41)	2367 (13.59)	1 [Reference]	NA	16640 (95.53)	779 (4.47)	1 [Reference]	NA	15757 (90.46)	1662 (9.54)	1 [Reference]	NA
40-64	8042 (79.62)	2059 (20.38)	1.71 (1.60-1.83)	<0.001	9787 (96.89)	314 (3.11)	0.81 (0.71-0.93)	0.003	8287 (82.04)	1814 (17.96)	2.09 (1.95-2.25)	<0.001
≥65	2181 (74.23)	757 (25.77)	2.38 (2.16-2.62)	<0.001	2868 (97.62)	70 (2.38)	0.69 (0.54-0.89)	0.004	2233 (76.00)	705 (24.00)	3.01 (2.72-3.33)	<0.001
Symptoms												
Asymptomatic	2952 (90.91)	295 (9.09)	1 [Reference]	NA	3183 (98.03)	64 (1.97)	1 [Reference]	NA	3012 (92.76)	235 (7.24)	1 [Reference]	NA
Symptomatic	22657 (81.84)	5028 (18.16)	2.47 (2.18-2.80)	<0.001	26566 (95.96)	1119 (4.04)	1.79 (1.39-2.32)	<0.001	23613 (85.29)	4072 (14.71)	2.63 (2.29-3.02)	<0.001
Wave												
1	2571 (84.54)	470 (15.46)	1 [Reference]	NA	2945 (96.84)	96 (3.16)	1 [Reference]	NA	2658 (87.41)	383 (12.59)	1 [Reference]	NA
2	12904 (84.02)	2454 (15.98)	1.31 (1.17-1.46)	<0.001	14671 (95.53)	687 (4.47)	1.50 (1.20-1.87)	<0.001	13498 (87.89)	1860 (12.11)	1.26 (1.12-1.42)	<0.001
3	10134 (80.86)	2399 (19.14)	1.64 (1.47-1.83)	<0.001	12133 (96.81)	400 (3.19)	1.43 (1.13-1.81)	0.003	10469 (83.53)	2064 (16.47)	1.67 (1.48-1.88)	<0.001

Abbreviations: aOR, adjusted odds ratio; NA, not applicable, OWT, onward transmission

[a] Factors associated with the generation of OWT were compared using multivariable logistic regression analysis adjusted for the history of visiting nightlife establishments, sex, age group, presence of symptoms, and wave. The absence of a history of visiting nightlife establishments, female sex, age 18 to 39 years, asymptomatic cases, and Wave 1 were set as references.

[b] Cases not generating OWT

[c] Cases generating OWT

© 2023 Imamura T et al. *JAMA Network Open.*
