# Supplementary material for: Transmission of COVID-19 in Nightlife, Household, and Health Care Settings in Tokyo, Japan, in 2020
Source: JAMA Netw Open. 2023 Feb 24;6(2):e230589. doi: 10.1001/jamanetworkopen.2023.0589 (PMC9958531; doi:10.1001/jamanetworkopen.2023.0589)
Supplement: Supplement 2. — Data Sharing Statement [file jamanetwopen-e230589-s002.pdf]

Data Sharing Statement

Imamura. Transmission of COVID-19 in Nightlife, Household, and Health Care Settings in Tokyo, Japan, in 2020. *JAMA Netw Open*. Published February 24, 2023. doi:10.1001/jamanetworkopen.2023.0589

Data
Data available: No

Additional Information
Explanation for why data not available: All the data analyzed in this study are not publicly available, and requests for accessing the data need to be directed to the Tokyo Metropolitan Government.
